# Metabolic profiling and pharmacokinetic studies of Baihu-Guizhi decoction in rats by UFLC-Q-TOF–MS/MS and UHPLC-Q-TRAP-MS/MS

**DOI:** 10.1186/s13020-022-00665-w

**Published:** 2022-10-04

**Authors:** Yan He, Zhenkun Zhou, Weijie Li, Yanqiong Zhang, Ruoyao Shi, Tao Li, Linlin Jin, Hongliang Yao, Na Lin, Hao Wu

**Affiliations:** 1 Guangdong Engineering and Technology Research Center for Quality and Efficacy Reevaluation of Post-Market Traditional Chinese Medicine, Guangdong Provincial Key Laboratory of Plant Resources, School of Life Sciences, Sun Yat-Sen University, No. 135, Xingangxi Street, Guangzhou, 510275 People’s Republic of China; 2 Institute of Chinese Materia Medica, China Academy of Chinese Medical Sciences, Beijin, China; 3 Guangdong Key Laboratory of Animal Conservation and Resource Utilization, Guangdong Public Laboratory of Wild Animal Conservation and Utilization, Institute of Zoology, Guangdong Academy of Sciences, Guangzhou, China

**Keywords:** Baihu-Guizhi decoction, Pharmacokinetics, Biotransformation, UHPLC-Q-TRAP-MS/MS, UFLC-Q-TOF-MS/MS

## Abstract

**Background:**

Baihu-Guizhi decoction (BHGZD) is a well-documented traditional Chinese Medicine (TCM) prescription that has been extensively applied to treating rheumatoid arthritis. Despite of its beneficial outcomes, the chemical constituents of BHGZD have not been fully portrayed and the in vivo absorption, distribution, metabolism, and excretion (ADME) patterns of absorbed components have never been described.

**Methods:**

Characterization of absorbed components and in vivo biotransformation profiling of these feature compounds were based on the ultra-fast liquid chromatography-quadrupole-time-of-flight tandem mass spectrometry (UFLC-Q-TOF-MS/MS). Furthermore, the ultra-high-performance liquid chromatography tandem ion trap quadrupole mass spectrometry (UHPLC-Q-TRAP-MS/MS) system were performed to investigate the pharmacokinetics of active ingredients from BHGZD.

**Results:**

In this study, we have identified and tentatively characterized 18 feature absorbed prototype and 15 metabolites of BHGZD in rat serum and the in vivo transformation pathways of these absorbed constituents were proposed. Besides, we have established novel quantitative methodology of five crucial components of BHGZD and have monitored the pharmacokinetic behaviors of these constituents spontaneously in rat serum after BHGZD gavage. After rats received two ways of BHGZD gavage, the pharmacokinetic behaviors of each compound exhibited relatively similar behaviors, as evidenced by similar curve track as well as relatively close time to reach maximum concentration (Tmax) and half washout time (T1/2). Whereas the maximum plasma concentration (Cmax) and area under the plasma concentration–time curve (AUC) values of five analytes with multiple dosage were a bit higher than single dosage.

**Conclusion:**

This study added knowledge into the material basis and bio-transformation patterns of BHGZD in vivo, which would be of great value for exploring pharmacological effects and mechanism of BHGZD.

**Supplementary Information:**

The online version contains supplementary material available at 10.1186/s13020-022-00665-w.

## Background

First reported in “Synopsis of the prescriptions of the golden chamber” (Chinese name: Jin Gui Yao Lue), Baihu-Guizhi Decoction (BHGZD) is one of the representative prescriptions of Zhang Zhongjing, the most eminent medical sage in traditional Chinese Medicine (TCM) practice. According to TCM theory, BHGZD is intended for heat repelling and Qi clearing, hence is perfectly suit the cases of Heat-invaded arthralgia. Clinically, BHGZD had been widely applied to treating Heat-characterized rheumatoid arthritis (RA) or osteoarthritis [1]. Mounting clinical evidences have emphasized the pharmacological effects of BHGZD in treating RA by alleviating synovial inflammation, hypertrophic synovium and bone destruction [2, 3]. However, although the pharmacological effects of BHGZD have been highly appreciated, the in vivo absorption, distribution, metabolism and excretion (ADME) behaviors of absorbed components of BHGZD have not yet been explored.

After taken orally, a drug underwent absorption and metabolism before it can reach its target tissue with sufficient abundance to exert bioactivities in vivo. Hence, for orally taken drugs, the comprehensive studies of its ADME patterns and behaviors in vivo is the prerequisite for in-depth deciphering its mechanism of actions. For TCM, however, medicinal herbs or ingredients are often used in combination as a formula. This makes TCM formula a sophisticated phytochemical system with abundant component unidentified and the in vivo ADME processes an intricate case to be explored. BHGZD, for example, is comprised of five medicinal ingredients including Gypsum Fibrosum (Shigao), *Anemarrhena asphodeloides* Bunge (Zhimu), *Neolitsea cassia* (L.) Kosterm. (Guizhi), *Oryza sativa* L. (Jingmi) and *Glycyrrhiza uralensis* Fisch. (Gancao) [4, 5]. Although the chemical composition of BHGZD had been tentatively characterized, and the speciation analysis of calcium contained in Baihu decoction was presented [6]. However, the in vivo ADME dynamics had never been explored, which greatly restrict the in-depth understanding of its in vivo absorption and mechanism explorations.

In the present study, we established a sensitive method to identify the absorbed components in vivo after oral administration of BHGZD by applying ultra-fast liquid chromatography-quadrupole-time-of-flight tandem mass spectrometry (UFLC-Q-TOF-MS/MS). And we proposed the in vivo metabolic pathways of chemical components from BHGZD. In addition, we conducted pharmacokinetic study of five absorbed components by using ultra-high-performance liquid chromatography tandem ion trap quadrupole mass spectrometry (UHPLC-Q-TRAP-MS/MS). To sum up, our study adds novel insight into the comprehensive understanding of the in vivo absorption and metabolic profiling of BHGZD, which would be useful in the interpretation for pharmacokinetics and pharmacodynamics of BHGZD in the future.

## Methods

### Chemicals and materials

Liquiritin apioside (purity: 96.0%) was purchased from Shanghai Standard Technology Co., Ltd. (Shanghai, China). Timosaponin AIII (purity: 98.7%), ononin (purity: 98.5%) and glycyrrhizic acid (purity: 99.6%) were obtained from Chengdu Must Bio-technology Co., Ltd. (Chengdu, China). Isoliquiritin (purity: 99.0%) was acquired from Chengdu DeSiTe Biotech Co., Ltd. (Chengdu, China). Mangiferin (purity: 98.0%), arginine (purity: 99.9%), citric acid (purity: 97.0%), isoleucine (purity: 99.9%), formononetin (purity: 98.0%), phenylalanine (purity: 99.9%) and cinnamic acid (purity: 98.8%) were provided by the National Institutes for the Control of Pharmaceutical and Biological Products (Beijing, China). Stable isotope-labeled [2',3',5',6'-D4]-naringin (D_4_-naringin, purity: 98.0%) was synthesized by Artis-chem Co., Ltd (Shanghai, China) as internal standards (IS).

MS grade methanol and acetonitrile were provided by Fisher Scientific Inc. (Fair Lawn, USA). Formic acid of MS grade was supplied by Sigma-Aldrich Co. (St. Louis, USA). Purified water was prepared by the Milli-Q system (Billerica, USA; electrical resistivity: 18.2 MΩ·cm) and then filtered through 0.22 μm microfiltration membrane before use. BHGZ decoction was provided by Institute of Chinese Materia Medica, China Academy of Chinese Medical Sciences (Beijing, China).

### Preparation of Baihu-guizhi decoction (BHGZD) powder

*Neolitsea cassia* (L.) Kosterm. (Guizhi), *Glycyrrhiza uralensis* Fisch. (Gancao), *Anemarrhena asphodeloides* Bunge (Zhimu), *Oryza sativa* L. (Jingmi) and Gypsum Fibrosum (Shigao) were authenticated and purchased from Beijing Tongrentang Co., Ltd (Beijing, China).

To prepare Baihu-Guizhi Decoction, Gypsum Fibrosum (60 g) was firstly extracted in 1,000 ml boiling water. After 30 min extraction, *Glycyrrhiza uralensis* Fisch. (5 g), *Neolitsea cassia* (L.) Kosterm. (10 g), *Anemarrhena asphodeloides* Bunge (15 g), *Oryza sativa* L. (30 g) was added to the aqueous for continuous extracted in boiling water for 30 min. Collected the first aqueous extract. Conducted the second extraction by immersing the residue in 800 ml boiling water for another 30 min and collected the second aqueous extract. Combined the extracts and concentrated to 300 ml volume. The BHGZD powder was obtained by drying out the concentrate in 70 ℃ oven overnight. Collected and weighed the powder. The final yield was 12 g crude materials per 1 g of BHGZD powder.

### Animals

Male Lewis rats, weighing 200–240 g, were purchased from Beijing Charles River Experimental Animals Co. Ltd. (Beijing, China). Animals were maintained under specific pathogen-free environment with constant temperature and humidity (22 ± 2 ℃ with 55 ± 15% relative humidity; 12 h light/dark cycle). Animal experiments were approved and supervised by the National Institutes of Health guide for the care and use of Laboratory animals. Every effort was made to minimize both the employed animal numbers as well as the potential discomfort of the animals throughout the study.

### Collection of rat serum

After 12 h food restriction, rats were subjected to single or multiple gavages of BHGZD. For single administration, rats were orally administrated with BHGZD powder at dose of 21.4 g/kg, which was equivalent to two-fold of clinical dose in human [7]. 200 μL venous blood was collected at 0 min, 15 min, 30 min, 1 h, 2 h, 3 h, 4 h, 6 h, 8 h, 12 h, 24 h post gavage. For multiple administration, rats were intragastrically administered BHGZD (21.4 g/kg/day) once a day, for continuous 12 days. On day 12, 200μL venous blood was collected at 0 min, 15 min, 30 min, 1 h, 2 h, 3 h, 4 h, 6 h, 8 h, 12 h, 24 h post gavage. Serum was freshly prepared by centrifugation and stored at – 80 ℃ till analysis.

### Preparation of stock solutions, calibration samples and quality control samples

To prepare stock solution, 10 mg liquiritin apioside, timosaponin AIII, glycyrrhizic acid, mangiferin, cinnamic acid and D4-naringin (IS) were accurately weighed, dissolved in methanol to a final concentration of 1.0 mg/mL. Working solutions of each reference standard were further prepared by a series of equimultiple dilutions of stock solutions, respectively.

To conduct calibration curve, a series of calibration solutions were prepared by mixing 10μL working solution at indicated concentrations with 10μL IS and 80μL blank rat serum to achieve desired concentration range: glycyrrhizic acid and cinnamic acid at 5, 25, 125, 250, 500 and 1000 ng/mL; mangiferin and liquiritin apioside at 1, 5, 25, 50, 100 and 200 ng/mL; timosaponin AIII at 0.5, 2.5, 12.5, 25, 50 and 100 ng/mL.

For method validation, QC samples were prepared at a high, medium, and low concentration with blank rat serum, with the desired concentrations of glycyrrhizic acid and cinnamic acid at 15, 150 and 750 ng/mL; mangiferin and liquiritin apioside at 3, 30 and 150 ng/mL; timosaponin AIII at 1.5, 15 and 75 ng/mL.

### Metabolic profiling of serum samples

To reveal the metabolic profiling of BHGZD in rat serum, a connected system of UFLC XR-hybrid triple quadruple time-of-flight mass spectrometer equipped with electrospray ionization source (ESI) was employed. Briefly, 100 μL serum was mixed with pre-cooled acetonitrile (containing 50 ng/mL IS) and vortexed vigorously for 2 min for protein precipitation. After centrifugation at 15,000*g* for 20 min, the supernatants were transferred and subjected to UFLC-Q-TOF-MS/MS for analysis.

Chromatographic separation was carried on a Kinetex C18 column (150 × 3.0 mm, 2.6 μm, Phenomenex, USA) using a Shimadzu UFLC XR system (Shimadzu, Japan), and mass detection on a 5600 plus quadrupole time-of-flight mass system (Triple TOF™ 5600 plus; AB Sciex, USA). The mobile phase was consisted of 0.1% aqueous formic acid (*v/v*; A) and methanol (B). The flow rate was 0.3 mL/min with the optimized gradient elution condition: 5%–95% B (0 − 40 min), maintained at 95% B for 5 min, 95%-5% B (45 − 50 min), followed by 2 min system equilibration. The flow rate was kept in 0.3 ml/min. The feature parameters of the mass spectrometer were described in our previously study [8]. Data analysis was processed using PeakView, Natural Products HR-MS/MS Spectral Library and MetabolitePilot software (all from AB Sciex, Forster City, USA).

### Quantification of absorbed compounds

Quantification for five absorbed compounds of BHGZD in rat serum were achieved by using a Shimadzu ultra-high-performance liquid chromatography tandem ion trap quadrupole Q-TRAP 6500 plus mass spectrometry (UHPLC-Q-TRAP-MS/MS). Sample preparations were described above.

Chromatographic separation was carried out on a Poroshell 120 EC-C18 column (50 × 3.0 mm, 2.7 μm, Agilent, USA) with a flow rate 0.3 mL/min. The mobile phases were 0.1% formic acid–water (*v/v*, A) and methanol (B) and corresponding gradient elution condition: 20%—40% B (0 – 0.5 min), maintained at 40% B for 0.5 min, 40%—90% B (1.0 – 3.5 min), maintained at 90% B for 1.5 min, 90%—20% (5.0 – 5.3 min) and 20% (5.3 – 8.0 min) for UHPLC system equilibration.

The optimized parameters for the mass spectrometer were set as follows: curtain gas, 35 psi; collision gas, medium; ionspray voltage, 4500 V; temperature, 550 °C; ion source gas 1 and 2, 55 psi. With the multiple reaction monitoring (MRM) detection, the information of five analytes and IS were optimized as shown in Table 1, including quantitative ion pairs, declustering potential and collision energy. Data acquisition and analysis were achieved by OS-Q software (version 1.4.1.20719, AB Sciex, USA). Pharmacokinetic analysis was conducted by Drug and Statistic software (version 3.0, Shanghai, China).

**Table 1:** The optimal MRM quantification parameters of five analytes and IS

Name	Precursor ion	Product ion	Declustering potential(V)	Collision energy(eV)
Timosaponin AIII	739.5	577.5	− 240	− 46
Glycyrrhizic acid	821.5	351.2	− 180	− 52
Liquiritin apioside	549.2	255.1	− 148	− 44
Mangiferin	421.2	301.1	− 63	− 30
Cinnamic acid	147.0	77.0	− 40	− 30
D_4_-naringin (IS)	583.2	275.1	− 212	− 44

### Quantification of calcium in Gypsum Fibrosum and BHGZD

Quantification for calcium were carried out by inductively coupled plasma atomic emission spectrometry (Optima 8300, PerkinElmer, USA). The parameters of ICP-AES were set as follows: incident power, 1300 W; plasma gas flow rate, 12L/min; nebulization gas flow rate, 0.55 L/min. Briefly, 1 mL Gypsum Fibrosum or BHGZD extraction was mixed with 5 mL concentrated nitric acid and nitrified 12 h. Then the solution was adjusted the volume to a 100 mL volumetric flask and subjected to ICP-AES for analysis.

## Results

### Identification of prototype and metabolites

Identifications of absorbent phytochemical components of BHGZD were achieved by chromatographic elution time, chemical composition, and feature fragmentation pattern in comparison with available reference standards and standard mass spectral library (Natural Products HR-MS/MS Spectral Library, Version 1.0, AB Sciex, Forster City, USA). With in-depth understandings of the distinct fragmentation rules of parent prototype skeletons, in vivo metabolites were tentatively characterized by integrating clues of chemical composition, product ions and fragmentation patterns. In this study, by using a highly sensitive UFLC-Q-TOF–MS/MS system, a total of 18 prototype phytochemicals and 15 metabolites were screened, both in positive and negative mode, in rat serum 0–24 h after BHGZD consumption (Additional file 1: Figure S1 and Additional file 2: Figure S2). Detailed information of compound description, chemical composition, retention time, mass error and characteristic fragmentation ions of BHGZD in vivo and in vitro are presented in Table 2 and Additional file 5: Table S1. And the calcium in Gypsum fibrosum and BHGZD were exhibited in Additional file 6: Table S2.

**Table 2:** Identification of absorbed components and metabolites in vivo after oral administration of BHGZD by UFLC-Q-TOF-MS/MS

No	Name	RT (min)	Formula	[M + H]^+^ (Error, ppm)	[M-H]^−^ (Error, ppm)	Characteristic Fragment ^a^	
						POS	NEG
P1	Arginine ^b^	1.94	C_6_H_14_N_4_O_2_	175.1187 (− 1.7)	ND	175.1176[M + H]^+^, 158.0911[M + H-NH_2_]^+^, 130.0952[M + H-HCOOH]^+^, 116.0683[M + H-NH_2_-CH_2_N_2_]^+^	ND
P2	Citric acid ^b^	2.44	C_6_H_8_O_7_	ND	191.0212 (7.6)	ND	191.0211[M-H]^−^, 173.0101[M-H-H_2_O]^−^, 129.0196[M-H-H_2_O-COOH]^−^, 111.009[M-H-H_2_O-COOH-H_2_O]^−^
P3	Isoleucine ^b^	2.44	C_6_H_13_NO_2_	132.1016 (− 2.7)	ND	132.1012[M + H]^+^, 86.0960[M + H-HCOOH]^+^, 69.0697[M + H-HCOOH-NH_3_]^+^	ND
P4	Phenylalanine ^b^	4.51	C_9_H_11_NO_2_	165.0789 (− 1.1)	164.0717 (5.7)	166.0847[M + H]^+^, 149.059[M + H-NH_2_]^+^, 120.0797[M + H-HCOOH]^+^, 103.0531[M + H-HCOOH-NH_2_]^+^	164.0706[M-H]^−^, 147.0433[M-H-NH_2_]^−^, 103.0536[M-H-COOH-NH_2_]^−^, 91.0522[M-H-COOH-NH_2_-CH]^−^
P5	Mangiferin ^b^	8.37	C_19_H_18_O_11_	423.0908 (− 3.2)	421.0779 (0.8)	423.0954[M + H]^+^, 369.0638[M + H-3H_2_O]^+^, 339.0489[M + H-3H_2_O-CH_2_O]^+^, 327.0471[M + H-C_5_H_2_O]^+^, 273.0394[M + H-C_5_H_10_O_5_]^+^, 257.0450[M + H-C_5_H_10_O_6_]^+^	421.0778[M-H]^−^, 403.0714[M-H-H_2_O]^−^, 331.0470[M-H-C_3_H_6_O_3_]^−^, 301.0353[M-H-C_4_H_8_O_4_]^−^, 259.0229[M-H-Glc]^−^, 243.0326[M-H-Glc-OH]^−^
M5-1	Methylation of P5	10.33	C_20_H_20_O_11_	437.1078 (− 4.3)	435.0933 (0.5)	437.1055[M + H]^+^, 383.0725[M + H-3H_2_O]^+^, 341.0686[M + H-C_5_H_2_O]^+^, 287.0592[M + H-C_5_H_10_O_5_]^+^,	435.0935[M-H]^−^, 345.0595[M-H-C_3_H_6_O_3_]^−^, 315.0495[M-H-C_4_H_8_O_4_]^−^, 272.0374[M-H-Glc]^−^
P6	7-*O*-Methyl Mangiferin ^b^	10.33	C_20_H_20_O_11_	437.1060 (− 4.3)	435.0933 (0.5)	437.1055[M + H]^+^, 419.0939[M + H-H_2_0]^+^, 317.0677[M + H-C_4_H_8_O_4_]^+^, 287.0592[M + H-C_5_H_10_O_5_]^+^	435.0935[M-H]^−^, 345.0595[M-H-C_3_H_6_O_3_]^−^, 315.0495[M-H-C_7_H_6_O_2_]^−^, 272.0374[M-H-Glc]^−^, 259.0230[M-H-Glc-CH_3_]^−^
M6-1	Glucuronidation of P6	7.47	C_26_H_28_O_17_	ND	611.1254 (8.8)	ND	611.1301[M-H]^−^, 435.0978[M-H-GluA]^−^
M6-2	Sulfation of P6	9.72	C_20_H_20_O_14_S	ND	515.0501 (1.3)	ND	515.0488[M-H]-, 435.0962[M-H-SO_3_]-, 345.0775[M-H-SO_3_-C_3_H_6_O_3_]^−^, 315.0520[M-H-SO_3_-C_7_H_6_O_2_]^−^
M6-3	Methylation of P6	14.25	C_21_H_22_O_11_	451.1235 (− 1.6)	449.1089 (1)	451.1261[M + H]^+^, 275.0899[M + H-Glc]^+^, 257.0817[M + H-Glc-H_2_O]^+^	449.1107[M-H]^−^, 273.0769[M-H-Glc]^−^, 255.0714[M-H-Glc-H_2_O]^−^
P7	Hydroxycinnamic acid	10.46	C_9_H_8_O_3_	165.0546 (11)	163.0454 (4.2)	165.0546[M + H]^+^, 147.0448[M + H-H_2_O]^+^, 119.0513[M + H-HCOOH]^+^, 91.0536[M + H-HCOOH-C_2_H_2_]^+^	163.0410[M-H]^−^, 119.0498[M-H-COOH]^−^, 93.0343[M-H-COOH-C_2_H_2_]^−^
P8	Liquiritin apioside ^b^	10.72	C_26_H_30_O_13_	568.2025 (− 2.7)	549.1782 (2.7)	568.4403[M + NH_4_]^+^, 419[M + H-C_5_H_9_O_4_]^+^, 257.0787[M + H-C_5_H_9_O_4_-C_9_H_6_O_3_]^+^	549.1620[M-H]^−^, 417.1096[M-H-C_5_H_9_O_4_]^−^, 255.0671[M-H-C_5_H_9_O_4_-C_9_H_6_O_3_]^−^
M8-1	Glucuronidation of P8	9.74	C_32_H_38_O_19_	ND	725.1928 (-0.9)	ND	725.1979[M-H]-, 549.1481[M-H-GluA]^−^
P9	Isoliquiritin ^b^	10.88	C_21_H_22_O_9_	ND	417.1187 (-0.9)	ND	417.1260[M-H]^−^, 255.0641[M-H-Glc]^−^, 135.0098[M-H-Glc-C_6_H_4_O-CO]^−^
M9-1	Glucuronidation of P9	7.92	C_27_H_30_O_15_	ND	593.1512 (3.3)	ND	593.1561[M-H]^−^, 417.1245[M-H-GluA]^−^, 255.0666[M-H-GluA-Glc]^−^
M9-2	Sulfation of P9	10.78	C_21_H_22_O_12_S	ND	497.0759 (1.7)	ND	497.0759[M-H]^−^, 417.1193[M-H-SO_3_]^−^, 254.9801[M-H-SO_3_-Glc]^−^
M9-3	Methylation of P9	17.69	C_22_H_24_O_9_	ND	431.1348 (4.3)	ND	431.1336[M-H]^−^
P10	Ononin ^b^	13.91	C_22_H_22_O_9_	431.1326 (-2.4)	ND	269.0792[M + H-Glc]^+^	ND
M10-1	Methylation of P10	14.11	C_23_H_24_O_9_	445.1493 (-5.7)	443.1348 (-1.1)	445.1391[M + H]^+^, 269.0970[M + H-Glc]^+^, 254.0726[M + H-Glc-CH_3_]^+^	443.1277[M-H]^−^, 267.0825[M-H-Glc]^−^, 252.0567[M-H-Glc-CH_3_]^−^
P11	Daidzein	14.74	C_15_H_10_O_4_	255.0647 (− 2.1)	253.0516 (3.8)	255.0643[M + H]^+^, 237.0527[M + H-H_2_O]^+^, 227.0708[M + H–CO]^+^	253.0514[M-H]^−^, 133.0273[M-H-C_7_H_4_O_2_]^−^
M11-1	Glucuronidation of P11	9.58	C_21_H_18_O_10_	431.0967 (− 1.3)	429.0826 (-0.2)	431.0918[M + H]^+^, 255.0652[M + H-Glc]^+^,	429.0801[M-H]^−^, 253.0508[M-H-GluA]^−^,
M11-2	Sulfation of P11	13.19	C_15_H_10_O_7_S	ND	333.0086 (3.5)	ND	333.0051[M-H]^−^, 253.0514[M-H-SO_3_]^−^
P12	Calycosin	15.22	C_16_H_12_O_5_	285.074 (− 5)	ND	285.0755[M + H]^+^, 270.0502[M + H-CH_3_]^+^	ND
M12-1	Glucuronidation of P12	9.88	C_22_H_20_O_11_	461.1062 (− 3.5)	ND	461.1024[M + H]^+^, 285.0748[M + H-GluA]^+^	ND
P13	Cinnamic acid ^b^	16.36	C_9_H_8_O_2_	149.0589 (− 5.6)	147.0508 (8.7)	149.0220[M + H]^+^, 131.0481[M + H-H_2_O]^+^, 103.0530[M + H-HCOOH]^+^, 77.0376[M + H-HCOOH-C_2_H_2_]^+^	147.0507[M-H]^−^, 103.0576[M-H-COOH]^−^, 77.0396[M-H-COOH-C_2_H_2_]^−^
P14	Timosaponin BIII ^b^	16.93	C_45_H_74_O_18_	903.4928 (− 2.2)	901.4842 (4.4)	903.4962[M + H]^+^, 741.4315[M + H-Glc]^+^, 579.3834[M + H-2Glc]^+^, 417.3382[M + H-2Glc-Gal]^+^, 399.3197[M + H-2Glc-Gal-H_2_O]^+^, 273.2187[M + H-2Glc-Gal-H_2_O-C_8_H_14_O]^+^, 255.2110[M + H-2Glc-Gal-H_2_O-C_8_H_15_O_2_]^+^	901.1910[M-H]^−^
P15	3',4',7-Trihydroxyisoflavone	17.55	C_15_H_10_O_5_	ND	269.0469 (5.2)	ND	269.0444[M-H]^−^, 133.0306[M-H-C_7_H_3_O_2_-H_2_O]^−^
M15-1	Glucuronidation of P15	11.57	C_21_H_18_O_11_	447.0907 (− 3.3)	445.0782 (1.3)	447.0908[M + H]^+^, 271.0608[M + H-GluA]^+^	445.0720[M-H]^−^, 269.0447[M-H-GluA]^−^
P16	Formononetin ^b^	19.85	C_16_H_12_O_4_	269.0808 (-− 2.9)	267.0682 (7.2)	269.0774[M + H]^+^	267.0667[M-H]^−^, 252.0415[M-H-CH_3_]^−^, 223.0446[M-H-H_2_O-CO]^−^
M16-1	Glucuronidation of P16	14.14	C_22_H_20_O_10_	445.1129 (− 0.8)	443.0984 (3.7)	445.1095[M + H]^+^, 269.0806[M + H-GluA]^+^	443.1020[M-H]^−^, 267.0678[M-H-GluA]^−^, 252.0433[M-H-GluA-CH_3_]^−^
M16-2	Sulfation of P16	18.72	C_16_H_12_O_7_S	ND	347.0231 (2.8)	ND	347.0258[M-H]^−^, 267.0662[M-H-SO_3_]^−^, 252.0447[M-H-SO_3_-CH_3_]^−^
P17	Glycyrrhizic acid ^b^	19.93	C_42_H_62_O_16_	ND	821.3997 (3.9)	ND	821.3990[M-H]^−^, 351.0582[M-H-C_30_H_46_O_4_]^−^
P18	Timosaponin AIII ^b^	25.26	C_39_H_64_O_13_	ND	785.4371 (5.3)	ND	785.4400[M-H]^−^, 739.4324[M-H-HCOOH]^−^, 577.3884[M-H-HCOOH-Glc]^−^

^a^The losses are: Glc = glucose moiety, GluA = glucuronyl moiety, Gal = galactose moiety, ND: not detected

^b^Confirmation in comparison with standard references

### Saponins and derived metabolites

3 saponins were firmly identified in rat serum after multiple gavages of BHGZD, among which two steroidal saponins, namely timosaponin BIII (P14) and timosaponin AIII (P18), were from medicinal herb *Anemarrhena asphodeloides* Bunge (Zhimu), the minister ingredient specialized for Heat repelling and Fire purging. Steroidal saponins are a group of remarkable active components of *Anemarrhena asphodeloides* Bunge. with bioactivities such as anti-inflammation [9] and tumor suppression [10]. In our study, the prototype of steroidal saponins timosaponin BIII (P14) and timosaponin AIII (P18) were identified after BHGZD gavage. These two saponins exhibited similar fragmentation behaviors including cleavage of glycosyl moiety, side chain as well as dehydration.

Another identified saponin of BHGZD in rat serum is glycyrrhizic acid (P17), one distinctive triterpene saponin from herb *Glycyrrhiza uralensis* Fisch. (Gancao). As one of the most studied active compounds of *Glycyrrhiza uralensis* Fisch., glycyrrhizic acid has been reported with potential activities such as anti-viral [11]. Moreover, due to the structural amphiphilic feature, glycyrrhizic acid could serve as a promising drug carrier to enhance the activity and bioavailability behaviors of other compounds, which might help to explain the adjuvant and coordinating properties of *Glycyrrhiza uralensis* Fisch. (Gancao) in the decoction [12].

### Flavonoids and derived metabolites

**Fig. 1:**
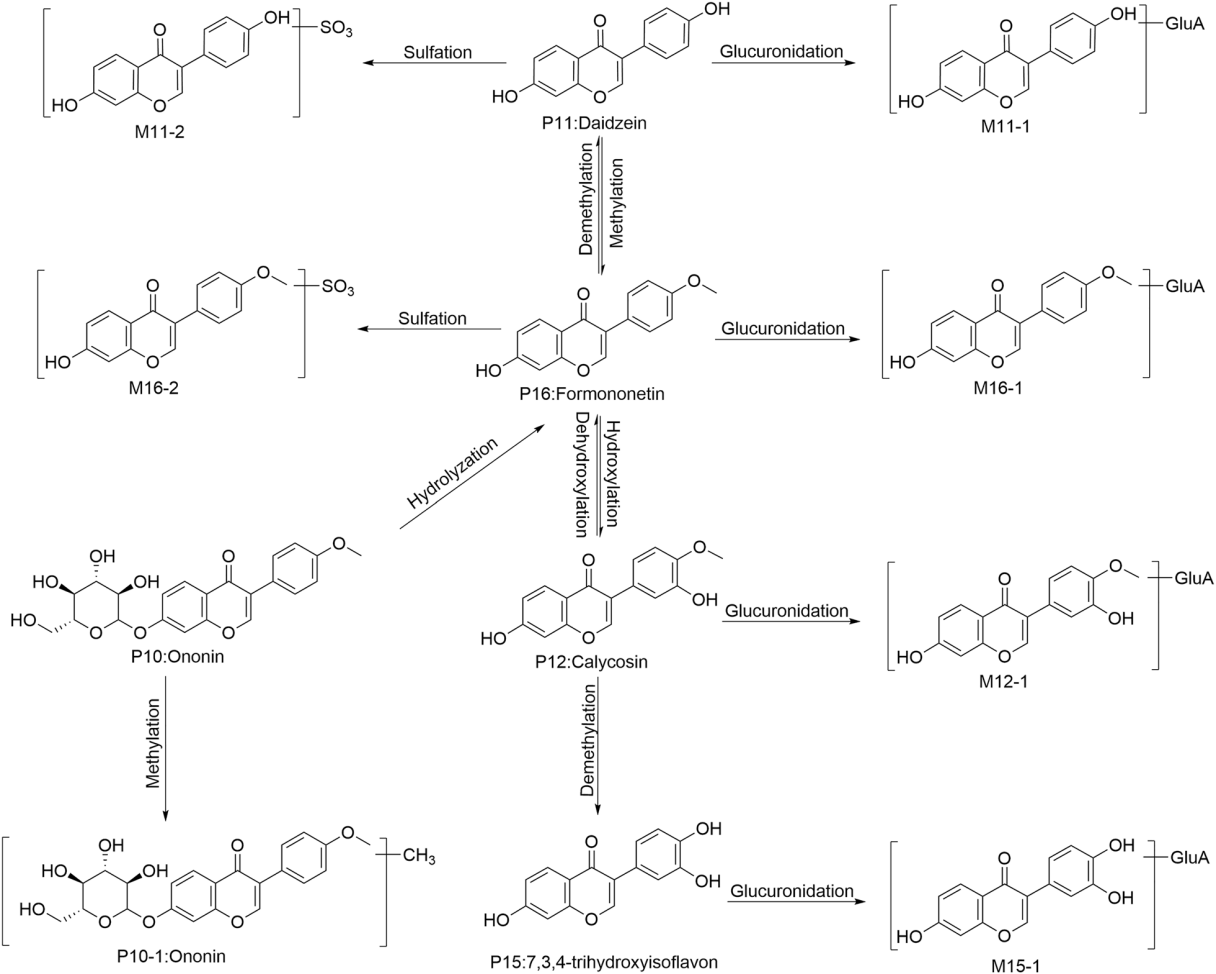
The metabolic pathways in vivo after oral gavage of BHGZD

9 flavonoids and 15 derived metabolites were identified or tentatively characterized after BHGZD gavage. For 9 prototype flavonoids, 6 of them, namely mangiferin (P5), 7-*O*-methyl mangiferin (P6), liquiritin apioside (P8), isoliquiritin (P9), ononin (P10) and formononetin (P16) were firmly identified while daidzein (P11), calycosin (P12) and 3',4',7-trihydroxyisoflavone (P15) were characterized in comparison with mass spectra library. Unlike saponins, these flavonoids underwent extensive phase I and II reactions in vivo. For example, ononin (P10) hydrolyzed (cleavage of Glc moiety) into its aglycone formononetin (P16). As for calycosin, demethylation happened when it loss a CH_3_ moiety and transform into 3',4',7-trihydroxyisoflavone, as evidenced by the loss of 15 Da of the precursor ion. For phase II reactions, sulfation was characterized by the loss of 80 Da SO_3_ moiety in mass spectrometry. In accord with this fragmentation pattern, M6-2, M9-2, M11-2 and M16-2 were tentatively identified as the sulfation metabolites of P6, P9, P11 and P16, respectively. Glucuronidation is another typical phase II reaction of flavonoids, which is identified by the characteristic GluA moiety. According to this structural feature, a series of glucuronidation metabolites of absorbent flavonoids, namely M6-1, M8-1, M9-1, M11-1, M12-1, M15-1, were tentatively identified with the loss of 176 Da GluA moiety of their precursor ions. By integrating the reactions mentioned above, we have proposed the comprehensive metabolic profiling of absorbent flavonoids of BHGZD (Fig. 1).

### Other components

Organic acids and amino acids were also absorbed in rat serum after BHGZD administration. Served as the minister ingredient of BHGZD, medicinal herb *Neolitsea cassia* (L.) Kosterm. (Guizhi) could effectively dispel exterior pathogen by diaphoresis induction. For *Neolitsea cassia* (L.) Kosterm. (Guizhi), it is well-accepted that the major effective components are grouped into volatile compounds such as cinnamaldehyde and soluble organic acids such as cinnamic acid, which accounted for the sweat inducing effect. In our study, for BHGZD, due to the water extraction preparation of the prescription, we are more focusing on the soluble part and have firmly identified hydroxycinnamic acid (P7) and cinnamic acid (P13) as active compounds of *Neolitsea cassia* (L.) Kosterm. (Guizhi). Moreover, 3 amino acids were characterized as arginine (P1), isoleucine (P3) and phenylalanine (P4), which may derive from ingredient *Oryza sativa* L. (Jingmi), the contribution of which to the overall beneficial outcomes of BHGZD should not be simply ignored.

### Methodology and pharmacokinetic analysis

**Fig. 2:**
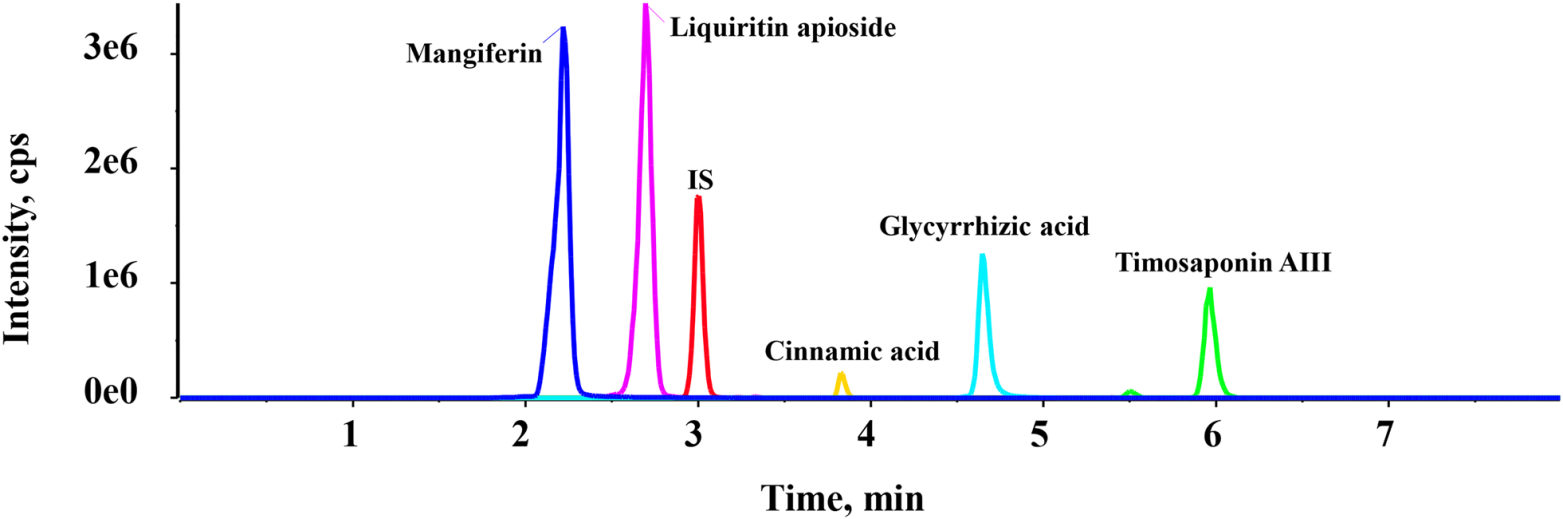
The optimized extracted ion chromatograms of five analytes in rat serum

Based on characterized absorbent components of BHGZD in rat serum, five absorbed prototypes (timosaponin AIII, glycyrrhizic acid, liquiritin apioside, mangiferin, cinnamic acid) were screened for further quantitative pharmacokinetic analysis. We have finetuned the chromatographic parameters such as column (Poroshell 120 EC-C18 column) and mobile phase system (0.1% formic acid water–methanol) for optimal separation. For each analyte, sharp peaks with satisfying resolutions were obtained (Fig. 2). Quantitative methodology was validated in accordance with the Guidance for Bioanalytical Method Validation (Chinese Pharmacopoeia, Version 2020). The selectivity of each analyte was shown in Additional file 3: Figure S3. Calibration curves, in corresponding coverage range of each analyte, were established with linear coefficients (r) > 0.99. For each compound, the precision of the limit of quantification (LLOQ) were less than 20%, the intra-run accuracy of low, middle and high quality control (QC) samples were within 15%, and matrix effects were all within 15% (Tables 3, 4, and 5). In brief, the methodology is practicable for quantitative analysis of these five compounds with acceptable linear coverage, precision, and accuracy.

**Table 3:** The regression equations, linear ranges five analytes after oral administration of BHGZD

Analytes	Range (ng/mL)	Linearity equations	r^2^
Timosaponin AIII	0.49 ~ 98.7	Y = 0.1853*X + 0.0015	0.9995
Glycyrrhizic acid	4.98 ~ 996	Y = 0.1597*X − 0.0212	0.9982
Liquiritin apioside	0.96 ~ 192	Y = 2.4322* X − 0.0040	0.9995
Mangiferin	0.98 ~ 196	Y = 1.2044*X + 0.0019	0.9992
Cinnamic acid	4.94 ~ 988	Y = 0.0859*X + 0.0039	0.9998

**Table 4:** The stabilities of five analytes in rat serum under different storage conditions

Analytes	Concentration (ng/mL)	Sample stability (mean ± SD, %)	Three freeze–thaw stabilities (mean ± SD, %)	4℃ short-term stabilities (mean ± SD, %)	− 80℃ long-term stabilities (mean ± SD, %)	room temperature short-term stabilities (mean ± SD, %)
Timosaponin AIII	1.48	91.95 ± 0.04	95.06 ± 0.02	100.7 ± 0.12	98.72 ± 0.19	96.95 ± 0.18
	74.0	96.37 ± 1.13	101.8 ± 2.03	96.19 ± 0.81	96.55 ± 4.29	99.54 ± 2.19
Glycyrrhizic acid	14.9	99.11 ± 0.74	97.89 ± 1.79	91.11 ± 0.56	97.61 ± 1.98	112.8 ± 0.12
	747	96.20 ± 5.96	102.0 ± 11.8	93.21 ± 9.08	100.38 ± 10.5	97.07 ± 3.97
Liquiritin apioside	2.88	100.9 ± 0.03	96.82 ± 0.08	99.05 ± 0.10	96.44 ± 0.01	100.4 ± 0.13
	144	105.4 ± 3.08	101.3 ± 0.15	99.5 ± 1.74	98.97 ± 3.63	98.40 ± 1.38
Mangiferin	2.94	90.85 ± 0.06	96.28 ± 0.18	99.46 ± 0.34	102.9 ± 0.32	94.09 ± 0.18
	147	106.2 ± 3.48	98.30 ± 2.40	97.90 ± 4.50	94.26 ± 8.31	98.02 ± 9.12
Cinnamic acid	14.8	110 ± 0.09	102.6 ± 1.21	109.7 ± 0.77	92.45 ± 0.79	98.33 ± 0.83
	741	102.8 ± 5.93	102.4 ± 3.94	96.63 ± 14.4	97.52 ± 4.26	98.67 ± 3.50

**Table 5:** The precision, accuracy and matrix effect of five analytes in rat serum (n = 5)

Analytes	Concentration (ng/mL)	Inter-day		Intra-day		Matrix effect
		RSD (%)	Accuracy RE (%)	RSD (%)	Accuracy RE (%)	RSD (%)
Timosaponin AIII	0.49	10.9	1.80	10.6	− 3.98	
	1.48	14.3	2.06	6.34	− 7.37	7.16
	14.8	6.79	− 2.70	11.9	− 3.88	
	74.0	2.81	− 6.26	9.55	− 4.58	1.65
Glycyrrhizic acid	4.98	7.25	2.45	8.34	− 6.05	
	14.9	8.17	0.74	13.8	− 3.00	9.01
	149	2.82	− 5.20	5.07	− 7.15	
	747	2.05	− 6.83	10.0	− 6.44	1.68
Liquiritin apioside	0.96	2.56	3.62	3.61	0.56	
	2.88	1.00	− 4.80	2.26	− 3.87	3.77
	28.8	2.38	− 2.37	3.41	− 4.67	
	144	2.07	− 1.50	3.50	− 3.44	1.76
Mangiferin	0.98	7.25	2.95	6.13	− 5.66	
	2.94	3.12	3.00	8.89	2.43	8.77
	29.4	2.06	− 4.08	5.54	− 3.93	
	147	5.55	− 1.27	5.00	3.80	3.83
Cinnamic acid	4.94	0.99	− 1.14	9.37	− 0.68	
	14.8	1.41	− 3.74	7.07	− 2.81	3.97
	148	2.45	− 0.95	3.07	− 3.62	
	741	0.84	− 1.11	2.25	− 0.59	2.32

**Fig. 3:**
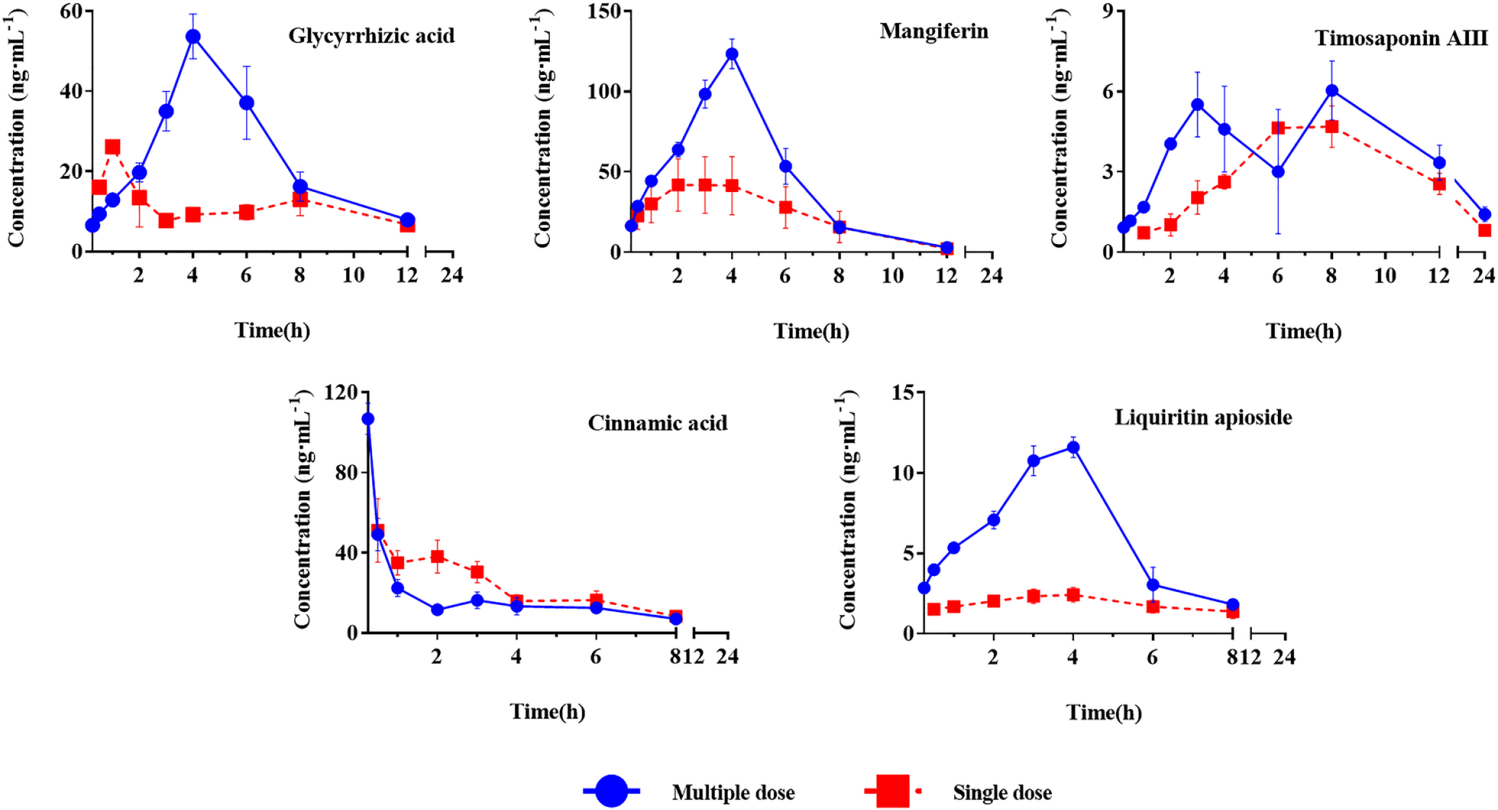
The serum concentration–time curves of five analytes in vivo after gavage with various dose BHGZD. (Blue line: multiple dose, red line: single dose, n = 8)

The developed methodology was then applied for the in vivo pharmacokinetic evaluations of these five absorbed components. After single or multiple gavages of BHGZD, rat serum was collected at indicated time points, and the concentrations of five analyte were quantified accordingly. Relevant pharmacokinetic parameters, including time to reach maximum concentration (T_max_), maximum concentration (C_max_), eliminated half-time (T_1/2_) and the area under curve (AUC), were calculated (Table 6). Meanwhile, the time-concentration dynamics of five compounds in rat serum were depicted (Fig. 3). As demonstrated, after multiple gavages of BHGZD, the pharmacokinetic behaviors of each compound exhibited relatively similar with that of single administration, as evidenced by similar curve track as well as relatively close T_max_ and T_1/2_. For most absorbed compounds, both with single or multiple gavages, had a relatively fast absorption and quick elimination in serum, except for one compound, timosaponin AIII, showed a dual-peak response with prolonged pattern of absorption and elimination. Of note, although continuous gavage had limited impact on the absorption dynamics of five absorbed components, it dramatically increased their serum abundance, both C_max_ and AUC showed significant extent of increases, ranging from approximately 1.5 to 4.5 folds higher.

**Table 6:** The pharmacokinetic parameters for five analytes in vivo after gavage with various dose BHGZD (n = 8, mean ± SEM)

Analytes	Times of administration	Timosaponin AIII	Glycyrrhizic acid	Liquiritin apioside	Mangiferin	Cinnamic acid
T_max_(h)	Single dose	6.38 ± 1.69	5.88 ± 2.85	4.00 ± 1.77	2.56 ± 1.68	0.37 ± 0.35
C_max_(ng/mL)		5.41 ± 1.23	15.61 ± 8.97	2.74 ± 1.23	48.10 ± 52.73	53.31 ± 32.64
T_1/2_(h)		4.98 ± 1.74	2.99 ± 0.82	2.65 ± 1.57	3.64 ± 4.82	2.36 ± 1.03
AUC(0–t)		35.56 ± 7.99	67.29 ± 36.83	11.771 ± 5.87	265.57 ± 305.54	109.70 ± 126.79
AUC(0–∞)		45.33 ± 13.95	91.78 ± 44.19	28.06 ± 9.12	297.72 ± 297.82	307.17 ± 119.68
MRT(0–t)		7.54 ± 1.15	5.06 ± 1.37	3.27 ± 0.80	3.60 ± 0.75	3.66 ± 1.03
MRT(0–∞)		9.92 ± 2.73	5.83 ± 1.42	12.17 ± 12.65	6.72 ± 6.64	10.53 ± 12.52
Vd(L/kg)		3,132,646 ± 264,876	867,518 ± 160,982	7,767,546 ± 4,149,931	1,049,685 ± 1,125,516	598,493 ± 614,714
CL(L/h/kg)		506,158 ± 133,767	288,330 ± 150,321	1,293,431 ± 1,002,413	228,709 ± 238,817	78,418 ± 31,121
T_max_(h)	Multiple dose	4.62 ± 2.13	4.38 ± 1.06	3.62 ± 0.51	3.88 ± 0.35	0.25 ± 0.01
C_max_ (ng/mL)		8.31 ± 1.32	58.22 ± 21.17	12.29 ± 2.01	126.74 ± 25.86	106.83 ± 21.78
T_1/2_ (h)		5.95 ± 2.19	2.48 ± 0.90	1.18 ± 0.44	1.27 ± 0.46	4.00 ± 2.81
AUC (0–t)		69.75 ± 15.18	280.22 ± 111.78	44.96 ± 10.19	538.35 ± 111.04	370.40 ± 46.18
AUC (0–∞)		76.17 ± 14.30	306.05 ± 112.46	47.91 ± 11.00	560.88 ± 135.88	443.26 ± 18.09
MRT(0–t)		9.52 ± 1.90	4.92 ± 0.64	3.15 ± 0.48	3.93 ± 0.49	9.64 ± 6.89
MRT(0–∞)		11.42 ± 2.50	5.85 ± 1.08	3.46 ± 0.69	4.15 ± 0.75	10.73 ± 5.54
Vd(L/kg)		2,313,233 ± 1,127,522	290,133 ± 197,532	718,240 ± 269,354	69,818 ± 16,069	455,197 ± 497,001
CL(L/h/kg)		289,940 ± 55,029	77,574 ± 25,214	470,072 ± 121,269	39,958 ± 8651	75,020 ± 55,222

## Discussion

To the best of our knowledge, this study firstly investigated the absorbed active constituents and unsealed the pharmacokinetic dynamics of multiple components of BHGZD in vivo. These results, when taken together, largely helped to decipher the absorption and metabolism of this classic decoction, which would be conducive for further in-depth mechanism explorations and rational application of BHGZD in clinical practice.

BHGZD is a well-known TCM prescription of medical sage Zhang Zhongjing for treating Heat-invaded symptoms such as arthralgia and arthritis. However, although the pharmacological effects of BHGZD have been highly appreciated, the in vivo absorption and metabolism of BHGZD have not been explored. In this study, we have identified the absorbed prototypes including saponins, flavonoids and organic acids, their derived metabolites and have depicted their biotransformation profiling comprehensively. Saponins tended to sustain prototype while flavonoids underwent intensive phase I/II metabolism. Meanwhile, by applying a poly-PK strategy, we have further monitored the in vivo dynamic behaviors of five crucial active components of BHGZD simultaneously. To sum up, the absorption and metabolic profiling in companion with quantitative dynamic evaluation could be of great significance for compound-activities interactions including safety and effectiveness investigation of BHGZD in the future.

For the quantitative PK study, we screened potential candidate compounds based on three major concerns. First of all, representative active compounds from predominant medicinal herbs should be included. For BHGZD, for instance, *Anemarrhena asphodeloides* Bunge (Zhimu) and *Neolitsea cassia* (L.) Kosterm. (Guizhi) are the minister ingredient which accounted for the effect of heat repelling and Qi clearing. To this concern, active compounds from these two herbs should be taken into serious consideration. For *Anemarrhena asphodeloides* Bunge, steroidal saponins and flavonoids are two major categories of active constituents, among which timosaponin AIII and mangiferin were the chose compounds of each category for subsequent PK study. Similarly, for *Neolitsea cassia* (L.) Kosterm. (Guizhi), one distinctive compound, cinnamic acid, was included. Secondly, the quantitative candidates should be presented in blood as prototypes with relative high abundances. In other words, the potential candidate should be technically traceable. If, for example, one compound showed extremely low blood concentration or can easily be eliminated, it is less possible to be included.

As one of the most commonly used medicinal herb for Heat-clearing and Fire-purging [13], *Anemarrhena asphodeloides* Bunge presented as minister ingredient of BHGZD with dominant dosage. Phytochemical studies had revealed that a group of steroidal saponins are the most significant bioactive components of *Anemarrhena asphodeloides* Bunge*,* which could help to explain the detoxicating and Heat-repelling properties of this herbal ingredient [14]. In this study, after BHGZD administration, we have detected two distinct steroidal saponins, timosaponin AIII and timosaponin BIII in rat serum with high abundances. Timosaponin AIII, in particular, had been reported with a variety of pharmacological activities, including tumor suppression [15], anti-inflammation [16] as well as inhibition of angiogenesis [17]. Hence, we further conducted the pharmacokinetics study of this compound and found a dual-peak absorption of timosaponin AIII in serum after gavage of BHGZD. This phenomeno would be partially caused by enterohepatic cycling, gastrointestinal emptying and the gut “absorption window” [18, 19]. To this concern, the dual-peak absorption property of timosaponin AIII should be taken into serious account when evaluating the in vivo time-dependent responses and pharmacological effects associated “time-window” of this compound.

*Neolitsea cassia* (L.) Kosterm. is a widely applied medicinal herb for collaterals dredging and pathogenic wind expelling with antipyretic properties. As a remarkable minister herb, *Neolitsea cassia* (L.) Kosterm. facilitates the heat-clearing effects of *Anemarrhena asphodeloides* Bunge and Gypsum Fibrosum, which ensures the beneficial outcomes of BHGZD against Heat syndrome related disorders and symptoms. In rat serum, we have identified cinnamic acid as major efficacious compounds of *Neolitsea cassia* (L.) Kosterm. Pharmacokinetic analysis revealed a rapid absorption pattern of cinnamic acid, with T_max_ approximately 0.3 h. Moreover, although cinnamic acid is quickly absorbed in the blood, it exhibits some extend of accumulative effects. The C_max_ of cinnamic acid with multiple BHGZD administration were approximately 2 times compared to single dose gavage, which indicated that for multi-component mixture, such as TCM formula, multiple delivery is a more rational and feasible strategy to increase the in vivo contents of active constituents.

In BHGZD, *Glycyrrhiza uralensis* Fisch. is the adjuvant ingredient with the major function of coordinating the actions of other ingredients of the prescription [20]. In this study, we have identified absorbed distinctive components of *Glycyrrhiza uralensis* Fisch. in rat serum, structurally categories into triterpene saponins and flavonoids. For triterpene saponins, glycyrrhizic acid was the most significant component with reported beneficial activities. Glycyrrhizic acid exerted potent anti-inflammatory roles [21], and could effectively alleviated hepatic damages in combined with liquiritin [22]. Despite of its own activities, glycyrrhizic acid had been reported to facilitate the membrane transfer of compounds and to enhance the bioavailability of certain components in vivo [12]. In other words, glycyrrhizic acid might be one of the crucial compounds contributed to the coordinative effects of *Glycyrrhiza uralensis* Fisch. by enhancing the serum contents of several other active compounds on one hand and hepatic toxicity-attenuating on the other hand.

Flavonoids are other group of active components that largely presented in *Glycyrrhiza uralensis* Fisch.. Over 32 *Glycytthiza* derived flavonoids had been characterized in BHGZD, among which 18 absorbed prototypes and their metabolites have been identified in our study. One particular flavonoid, ononin, caught our special attention. As a promising agent against inflammation [23] and angiogenesis [24], ononin had been widely recognized for its board range of metabolic transformation in vivo. Once taken orally, ononin was first cleavage by gut bacterial derived β-glycosidase into its aglycone, formononetin [25]. Subsequently, formononetin went through intensive phase I and II metabolism such as sulfation and glucuronidation [26]. Moreover, formononetin could reversibly be transformed into another active flavonoid calycosin via hydroxylation or dihydroxylation [27]. These together emphasized the significance of flavonoids related metabolic profiling. Special attentions should be made to comprehensively uncover the in vivo metabolic network of certain flavonoids, as these complex metabolic patterns indeed played non-negligible impacts on the in vivo pharmacological effects of prototypes.

Gypsum Fibrosum acted as sovereign drug in BHGZD and mainly consisted of poorly-soluble CaSO4. In addition to in vitro ICP-AES quantitative studies for BHGZD, we haven’t identified any Gypsum Fibrosum derived components owing to the limitations of the current instrument technology. Although CaSO4 is hardly dissolved in water, small amount of Ca^2+^ might be extracted and took part in bioactivities in a manner of secondary messenger or by interacting with calmodulins or increasing Ca^2+^ content [28, 29].

Favorable pharmacokinetic behaviors ensure drug-like properties. For TCM with multi-component nature, understandings of the in vivo absorption profiling, metabolic networks as well as dynamic patterns of active constituents are the prerequisite to build the potential association between effective compounds and the beneficial outcomes of the complex TCM formula. The absorbed components, including both bioactive prototypes as well as their metabolites, of prescription reach proper blood concentration to exert effects, so as BHGZD. However, the complexity property of TCM on human health make the evaluation of the effectiveness and safety and the clarification of underlying mechanism very challenging. Integrating these two parts allows us to create a whole scene of the in vivo behaviors of multiple components from BHGZD, which may help to unscramble the in vivo absorption and pharmacodynamic mechanism of actions of BHGZD in future exploration. Our strategy established in the study applies both absorbed profiles and metabolic transformation, which enable us to identify key active compounds exposed to serum and explored potential active metabolites. The advance of the poly-PK strategy will help to uncover the potential association between multi-compounds and the in vivo metabolic pathways and greatly facilitate the effectiveness evaluation of BHGZD candidates and novel clinical application.

## Conclusions

For TCM study, a comprehensive understanding of the in vivo behaviors of feature components is a prerequisite for further in-depth pharmacological and mechanism exploration. Establishing strategy for fully unseal the in vivo absorption, bio-transformation as well as pharmacokinetics of multicomponent systems, such as TCM, is a long-lasting bottleneck. In this study, taking BHGZD as a typical prescription, we have unsealed the in vivo absorption profiling and proposed metabolism network of this TCM formula. We have monitored the kinetic parameters of several key components of BHGZD simultaneously with a poly-pharmacokinetics strategy. Integrating these two parts allows us to create a whole scene of the in vivo behaviors of multiple components from BHGZD, which may help to unscramble the efficacy-associated mechanism of actions of BHGZD in future exploration.

## Supplementary Information

**Additional file 1: Figure S1.** The chemical structure and corresponding mass spectrum of absorbed components in vivo.

**Additional file 2: Figure S2.** The chromatograms of mixed standards and serum sample after BHGZD gavage.

**Additional file 3: Figure S3.** The chromatogram of selectivity of the analytes in rat serum.

**Additional file 4: Figure S4.** Graphical abstract.

**Additional file 5: Table S1.** Identification of chemical profile of BHGZD in vitro by UFLC-Q-TOF-MS/MS.

**Additional file 6: Table S2.** Quantification analysis of calcium in Gypsum Fibrosum and full prescription BHGZD by ICP-AES.

## Data Availability

The datasets supporting the conclusions of this article are included within the article and its additional files.

## Abbreviations

ADME: Absorption, distribution, metabolism, and excretion
AUC: Area under the plasma concentration–time curve
BHGZD: Baihu-Guizhi Decoction
Cmax: Maximum concentration
RSD: Relative standard deviation
Tmax: Time to reach maximum concentration
TCM: Traditional Chinese Medicine
t1/2: Half washout time
UFLC-Q-TOF-MS/MS: Ultra-high-performance liquid chromatography tandem ion trap quadrupole mass spectrometry
UHPLC-QTRAP-MS/MS: Ultra-high-performance liquid chromatography tandem ion trap quadrupole mass spectrometry
