# Supplementary material for: Metabolic profiling and pharmacokinetic studies of Baihu-Guizhi decoction in rats by UFLC-Q-TOF–MS/MS and UHPLC-Q-TRAP-MS/MS
Source: Chin Med. 2022 Oct 4;17:117. doi: 10.1186/s13020-022-00665-w (PMC9531372; doi:10.1186/s13020-022-00665-w)
Supplement: Supplementary file 1 — Additional file 1: Figure S1. The chemical structure and corresponding mass spectrum of absorbed components in vivo. [file 13020_2022_665_MOESM1_ESM.pdf]

Figure S1. The chemical structure and corresponding MS/MS spectrum of absorbed components *in vivo.*

Chemical structure	Negative mode	Positive mode

P1 Arginine

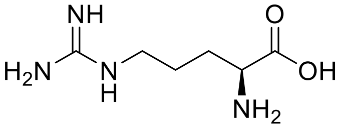

Negative mode: **ND**

Positive mode:

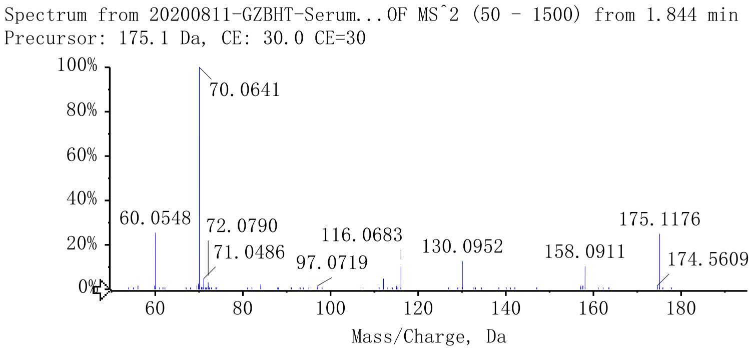

P2 Citric acid

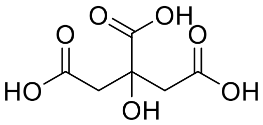

Negative mode:

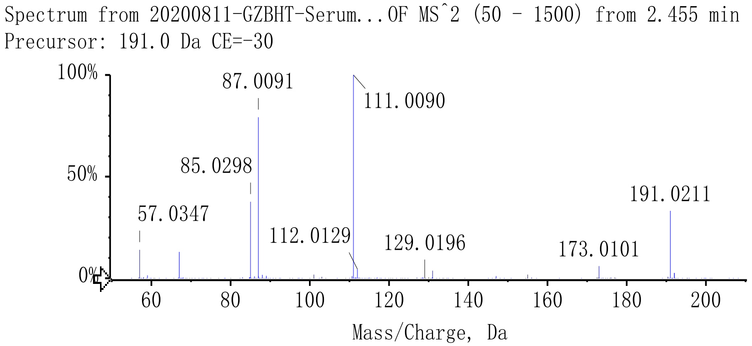

Positive mode: **ND**

P3 Isoleucine

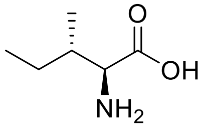

Negative mode: **ND**

Positive mode:

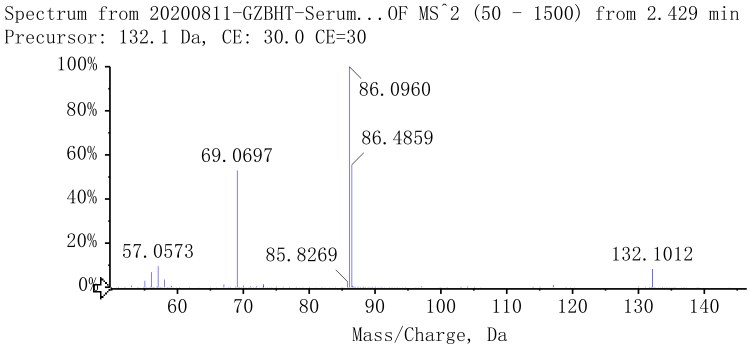

Figure S1. The chemical structure and corresponding MS/MS spectrum of absorbed components *in vivo.*

Chemical structure	Negative mode	Positive mode

P4 Phenylalanine

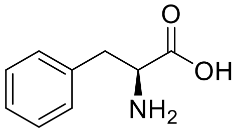

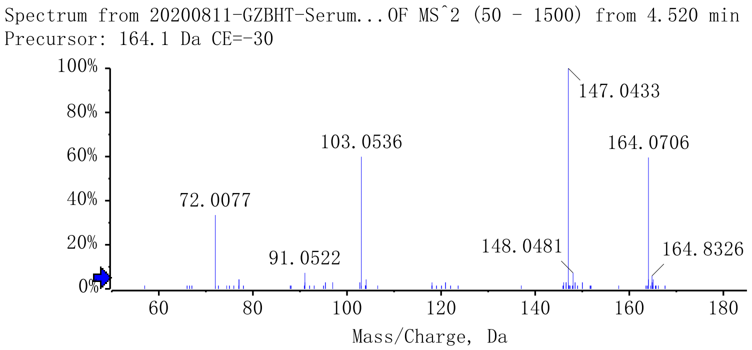

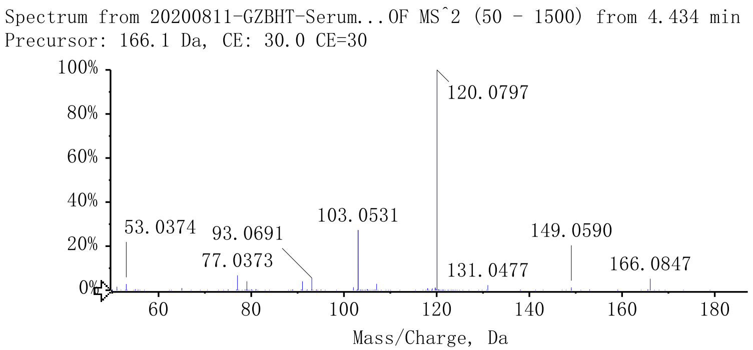

P5 Mangiferin

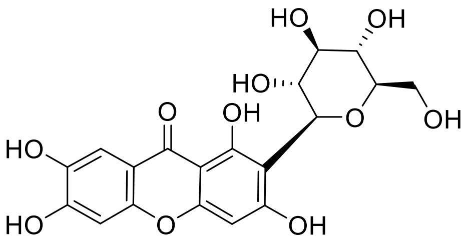

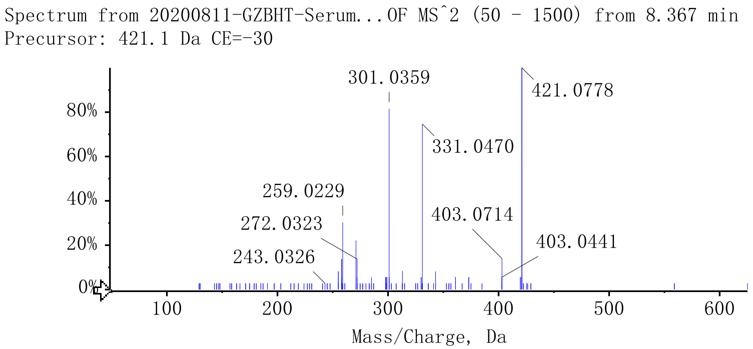

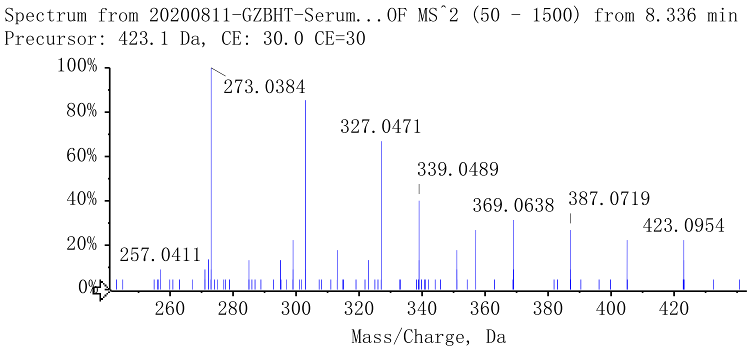

P5-1 Methylation of P5

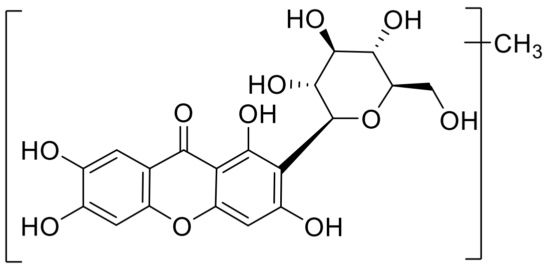

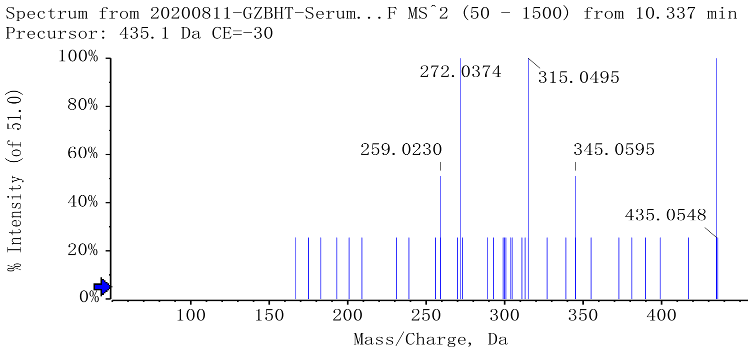

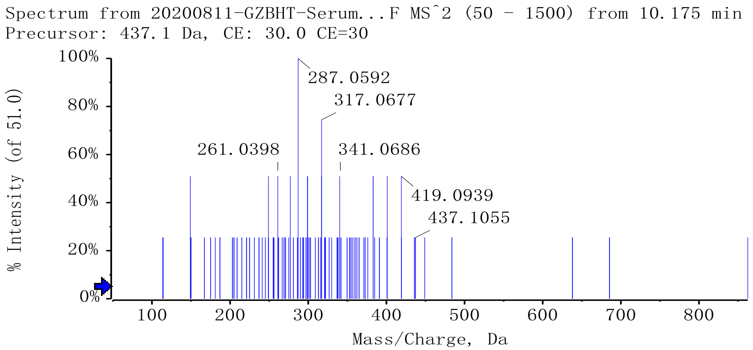

Figure S1. The chemical structure and corresponding MS/MS spectrum of absorbed components *in vivo.*

Chemical structure	Negative mode	Positive mode

P6 7-*O*-Methyl Mangiferin

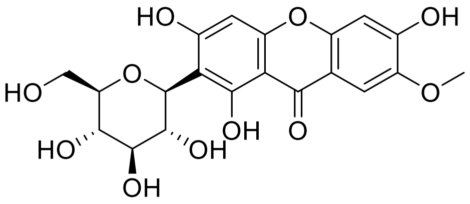

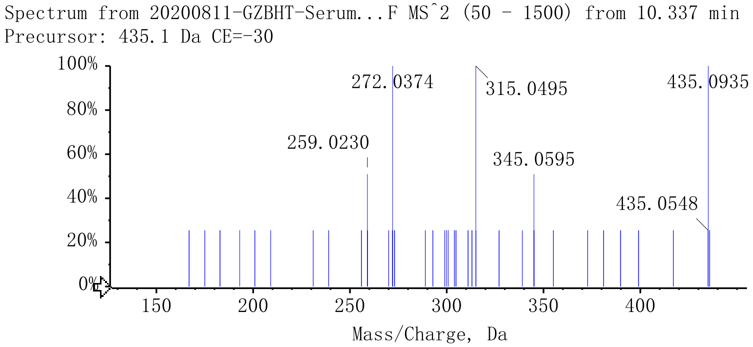

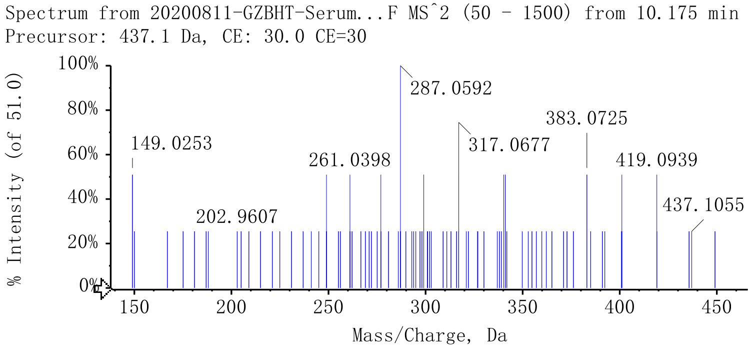

P6-1 Glucuronidation of P6

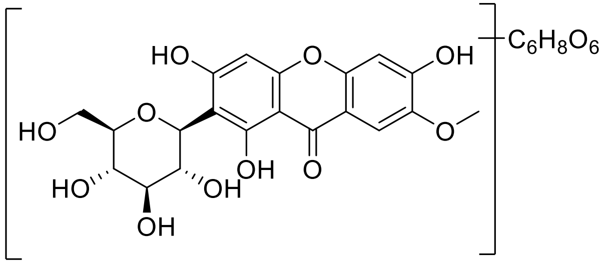

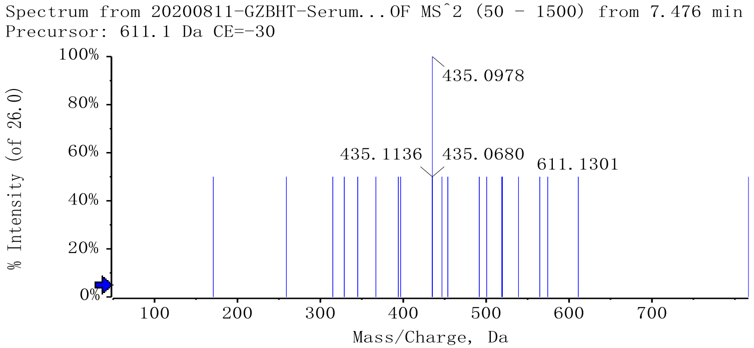

ND

P6-2 Sulfation of P6

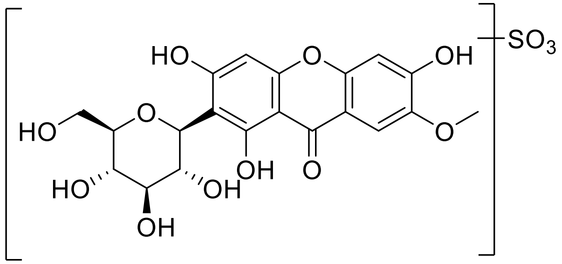

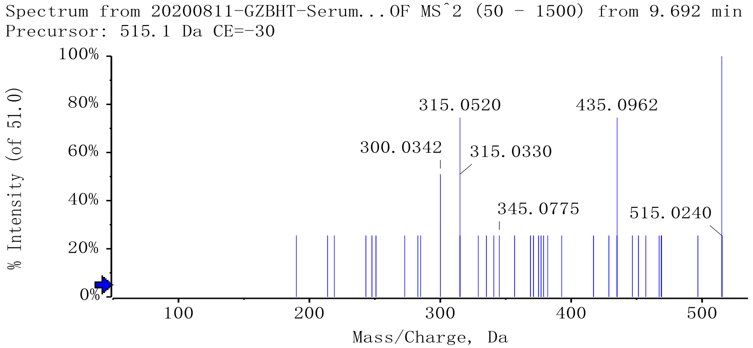

ND

Figure S1. The chemical structure and corresponding MS/MS spectrum of absorbed components *in vivo*.

**Chemical structure**	**Negative mode**	**Positive mode**

P6-3 Methylation of P6

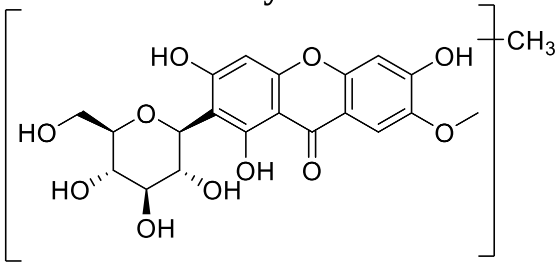

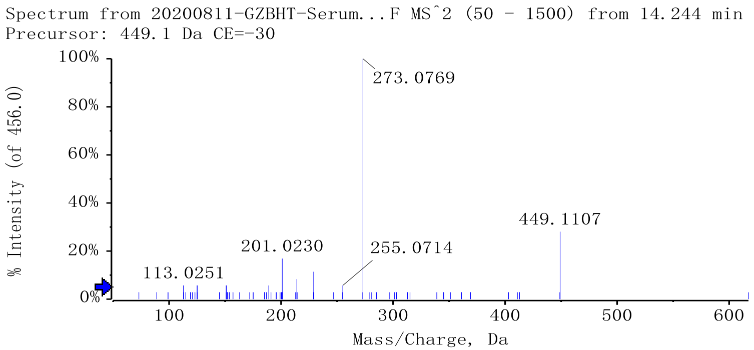

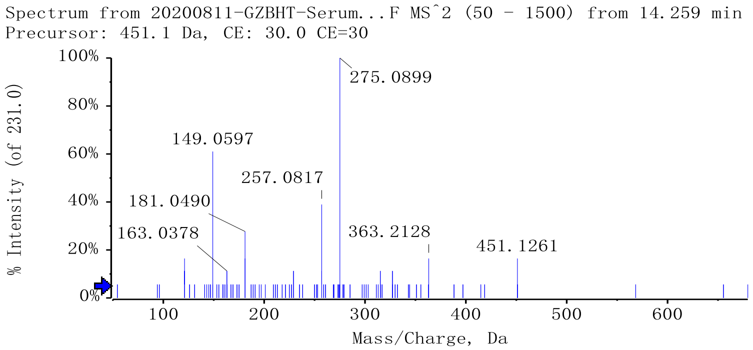

P7 Hydroxycinnamic acid

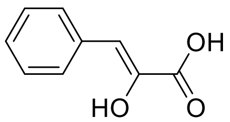

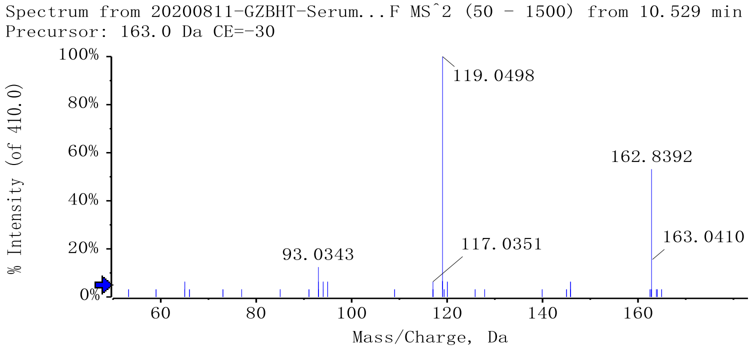

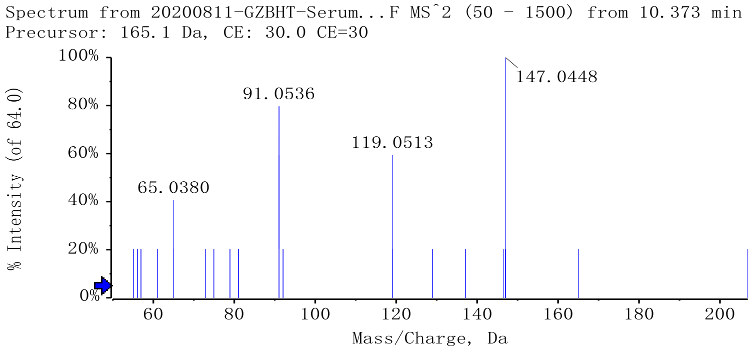

P8 Liquiritin apioside

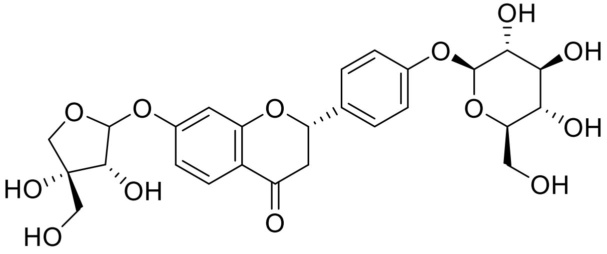

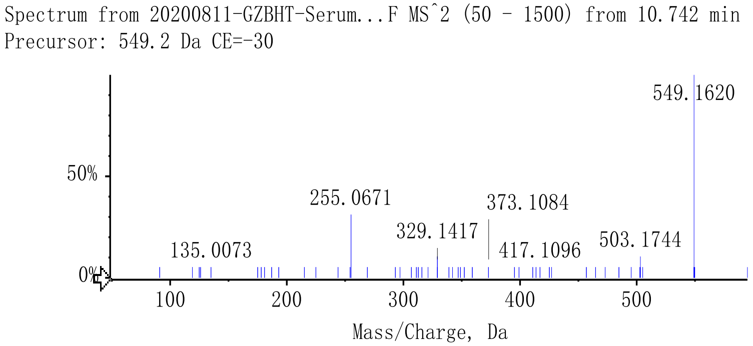

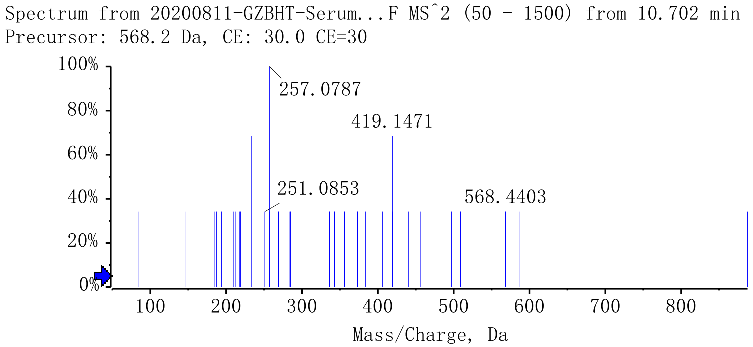

Figure S1. The chemical structure and corresponding MS/MS spectrum of absorbed components *in vivo.*

Chemical structure	Negative mode	Positive mode
**P8-1 Glucuronidation of P8** 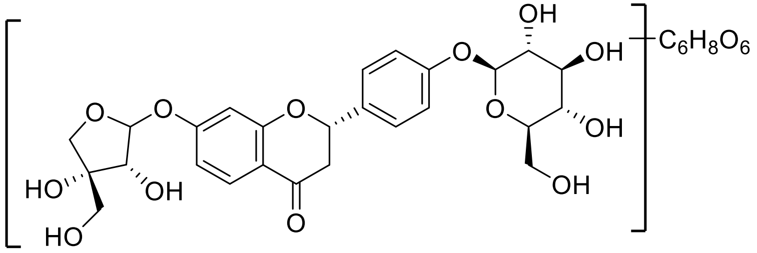	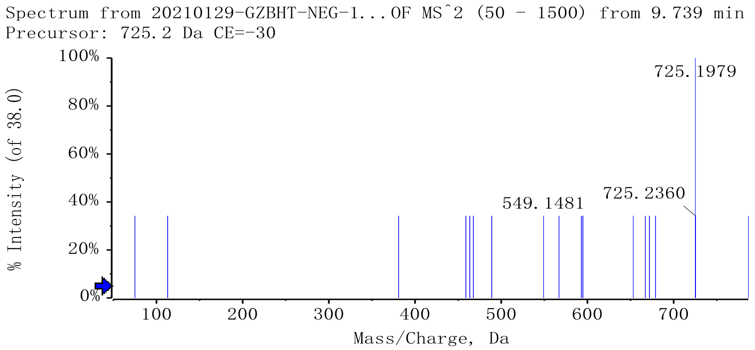	**ND**
**P9 Isoliquiritin** 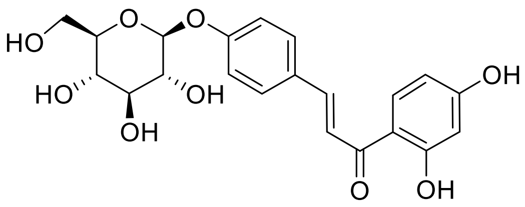	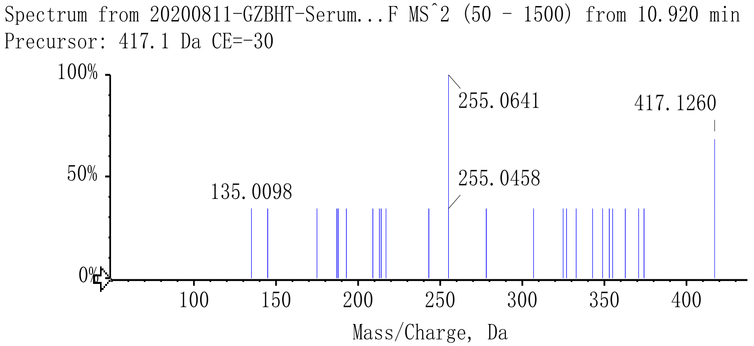	**ND**
**P9-1 Glucuronidation of P9** 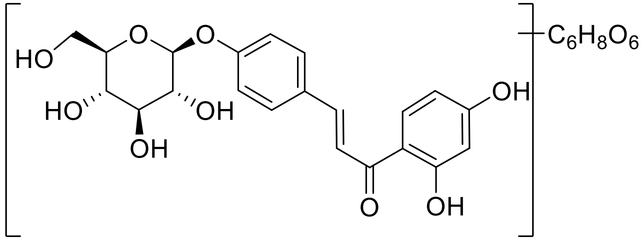	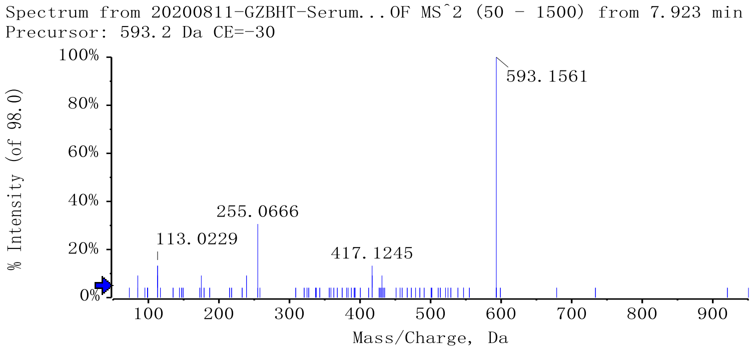	**ND**

Figure S1. The chemical structure and corresponding MS/MS spectrum of absorbed components *in vivo.*

Chemical structure	Negative mode	Positive mode

P9-2 Sulfation of P9

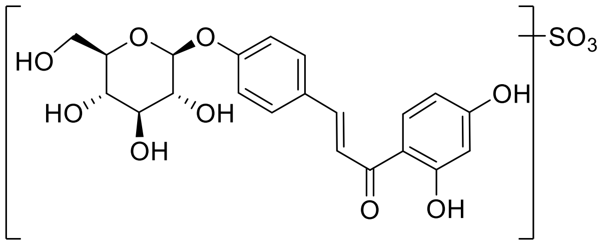

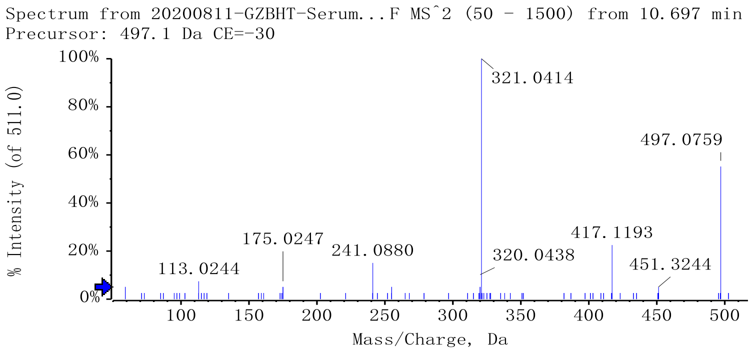

Positive mode: **ND**

P9-3 Methylation of P9

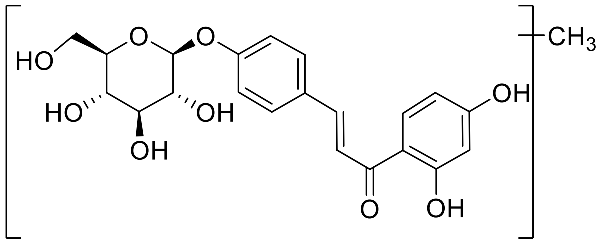

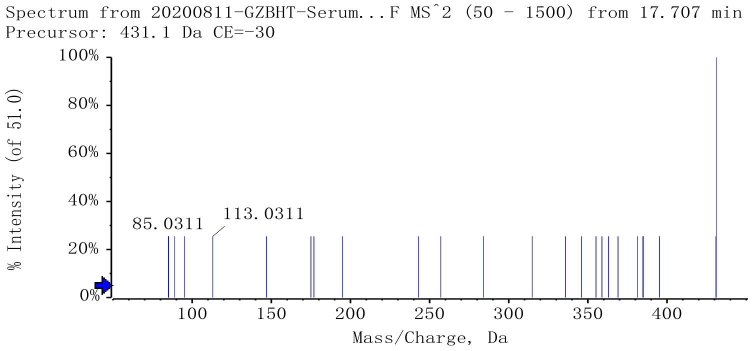

Positive mode: **ND**

P10 Ononin

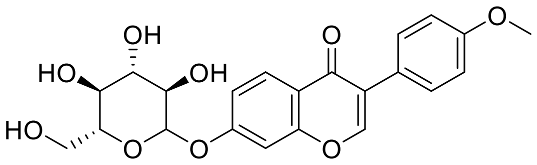

Negative mode: **ND**

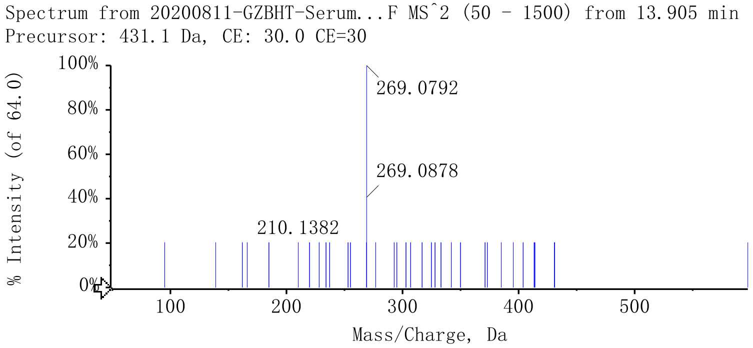

Figure S1. The chemical structure and corresponding MS/MS spectrum of absorbed components *in vivo.*

Chemical structure	Negative mode	Positive mode

P10-1 Methylation of P10

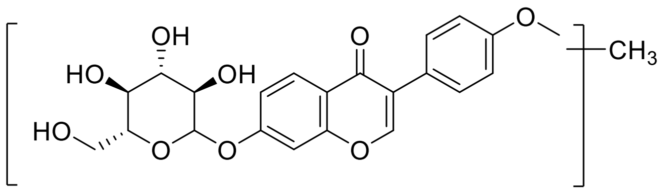

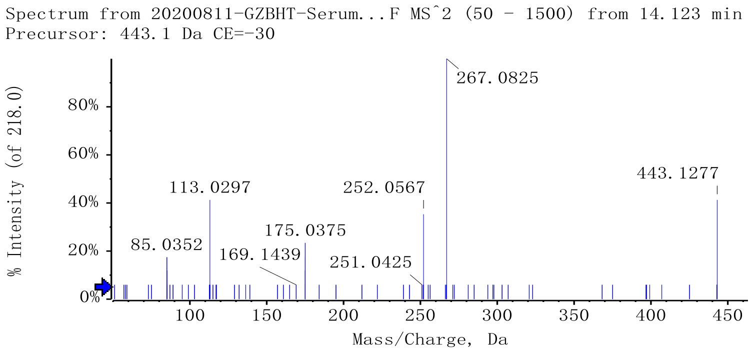

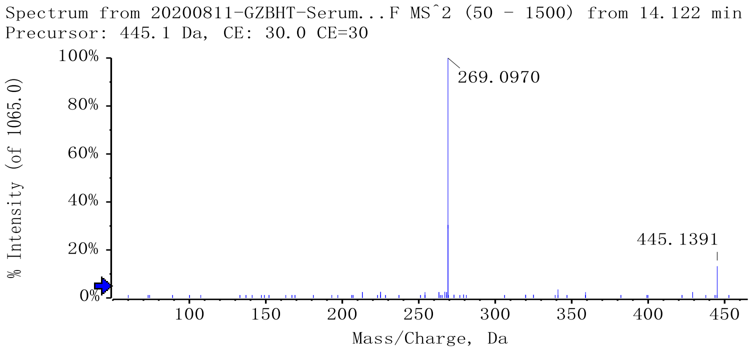

P11 Daidzein

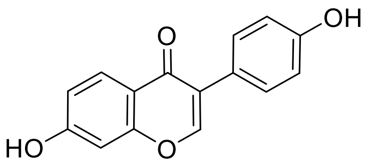

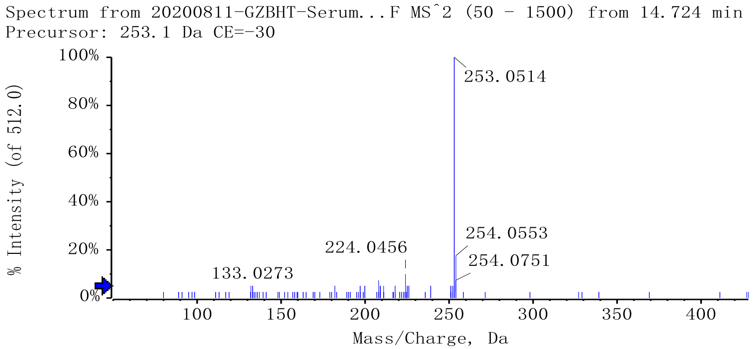

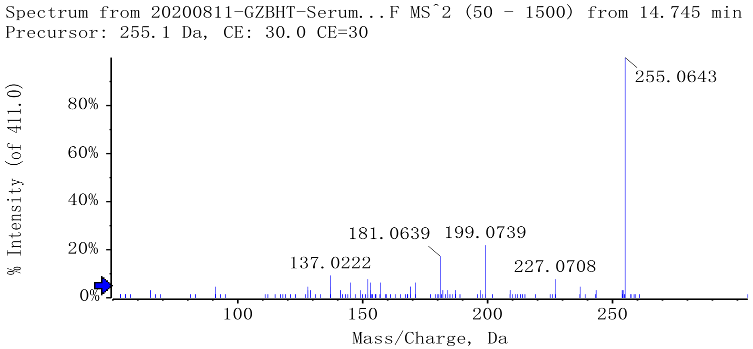

P11-1 Glucuronidation of P11

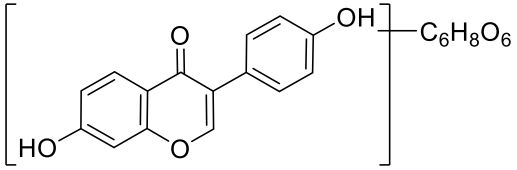

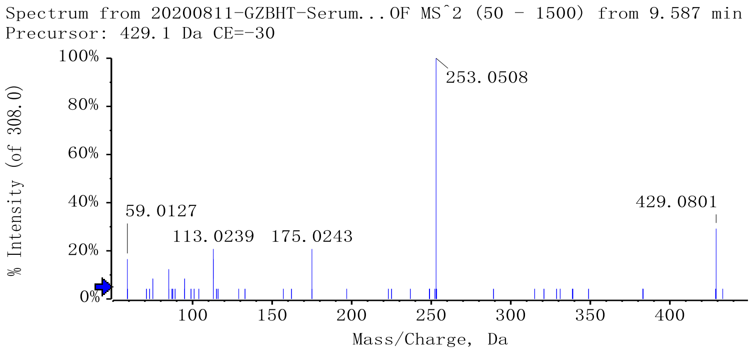

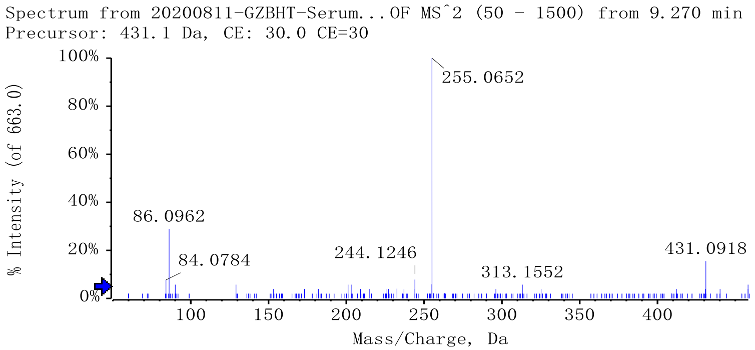

Figure S1. The chemical structure and corresponding MS/MS spectrum of absorbed components *in vivo.*

Chemical structure	Negative mode	Positive mode
**P11-2 Sulfation of P11** 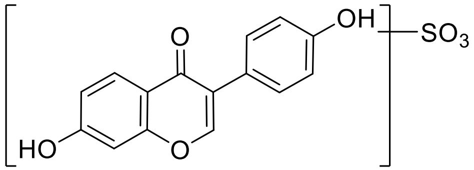	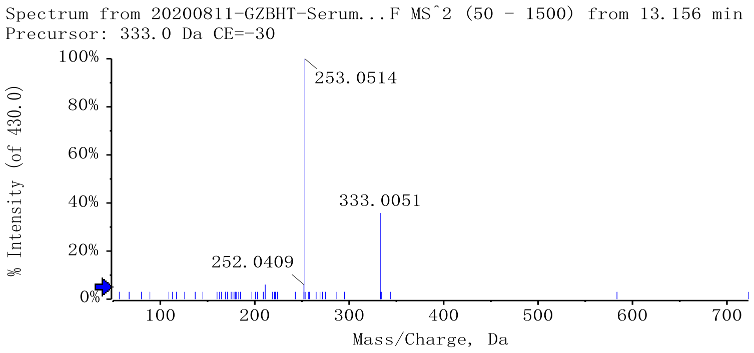	**ND**
**P12 Calycosin** 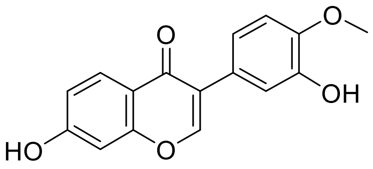	**ND**	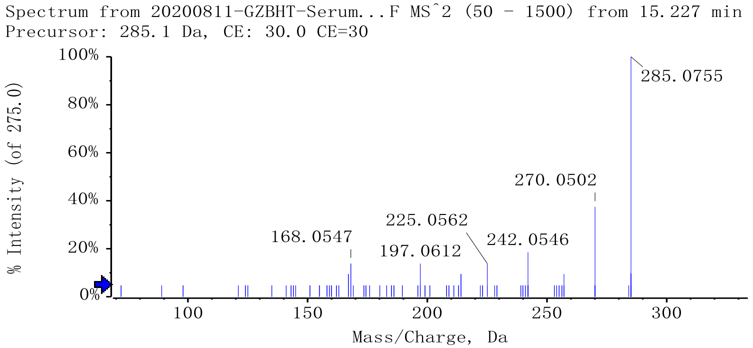
**P12-1 Glucuronidation of P12** 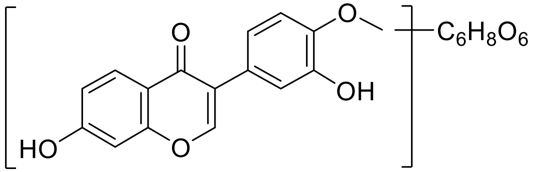	**ND**	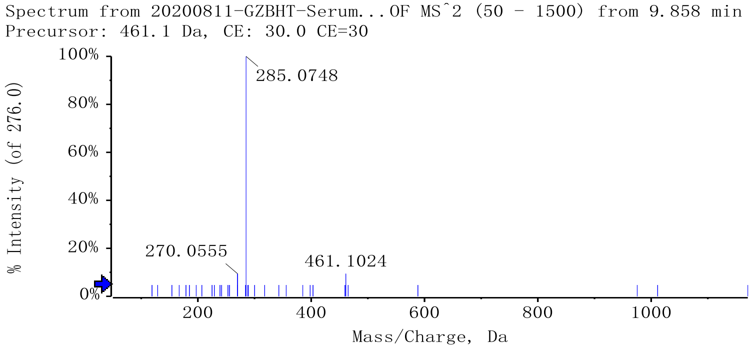

Figure S1. The chemical structure and corresponding MS/MS spectrum of absorbed components *in vivo.*

Chemical structure	Negative mode	Positive mode

P13 Cinnamic acid

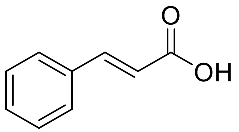

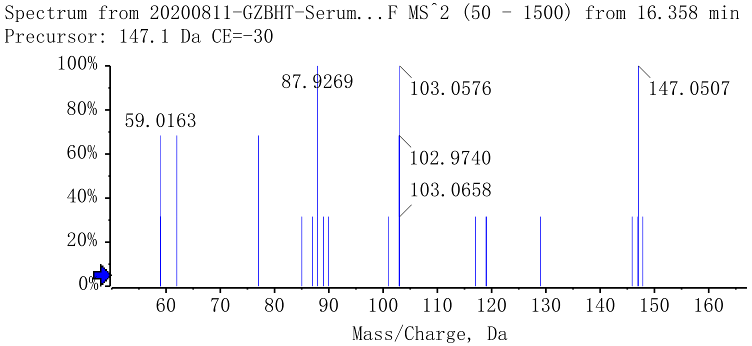

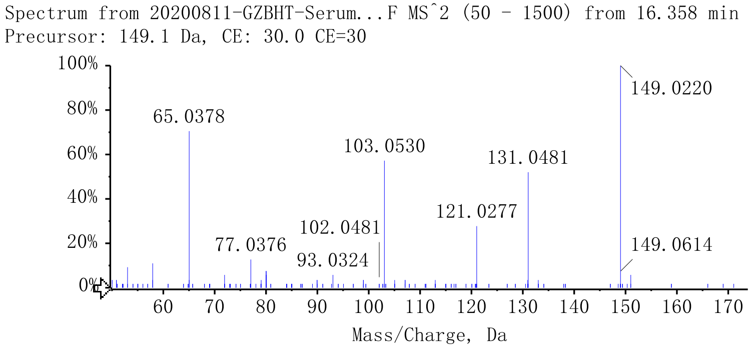

P14 Timosaponin BIII

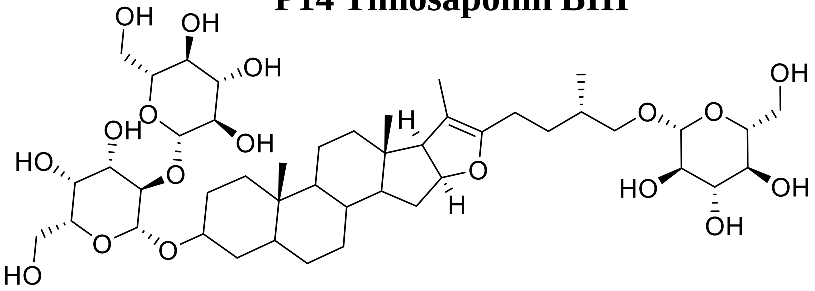

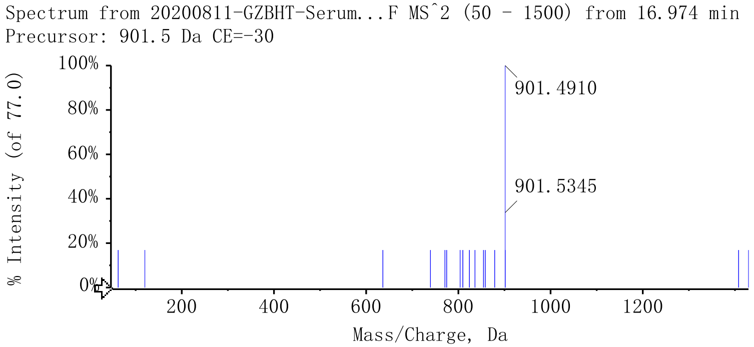

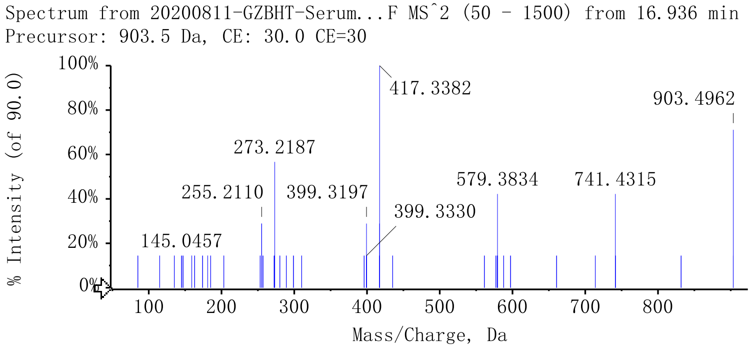

P15 3',4',7-Trihydroxyisoflavon

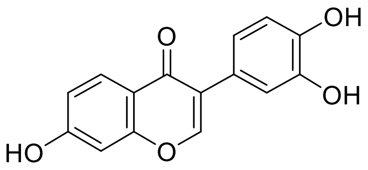

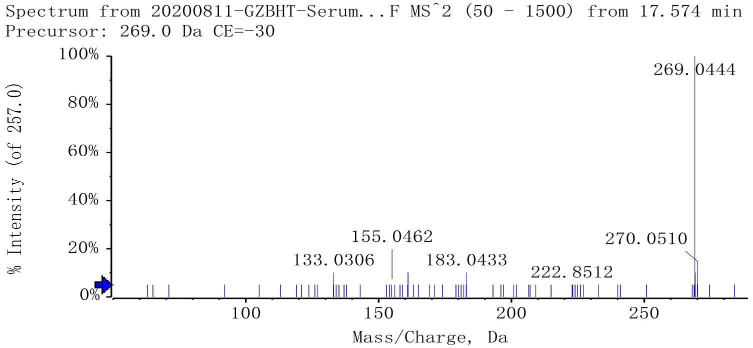

ND

Figure S1. The chemical structure and corresponding MS/MS spectrum of absorbed components *in vivo*.

Chemical structure	Negative mode	Positive mode

P15-1 Glucuronidation of P15

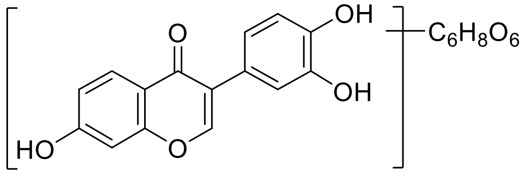

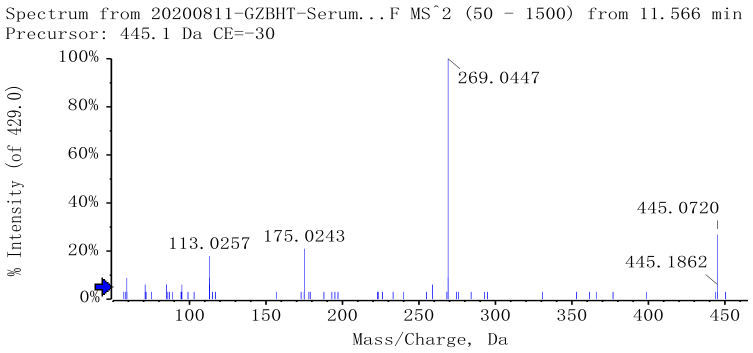

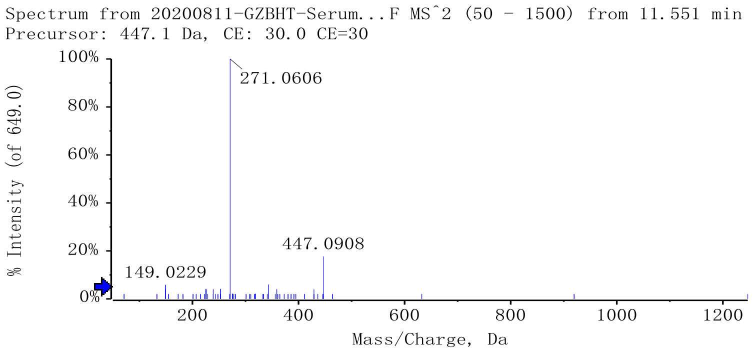

P16 Formononetin

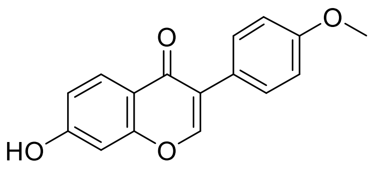

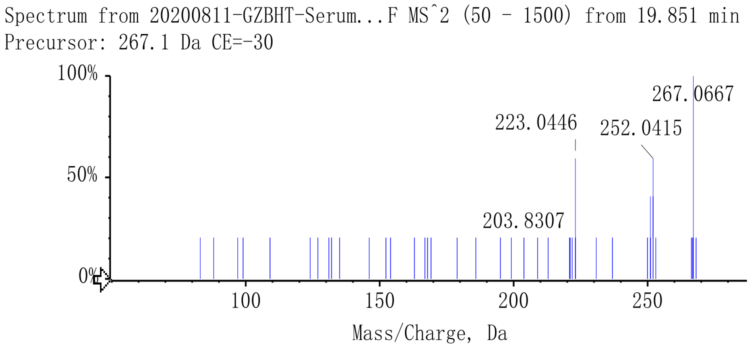

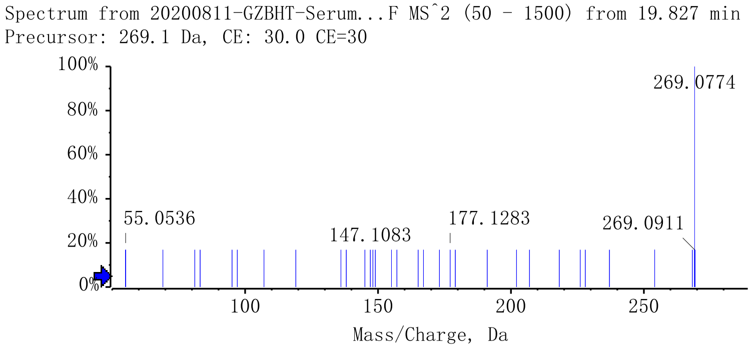

P16-1 Glucuronidation of P16

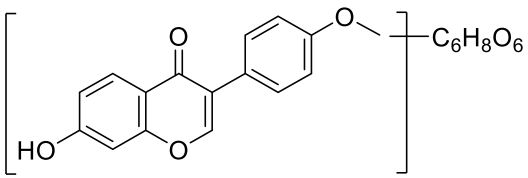

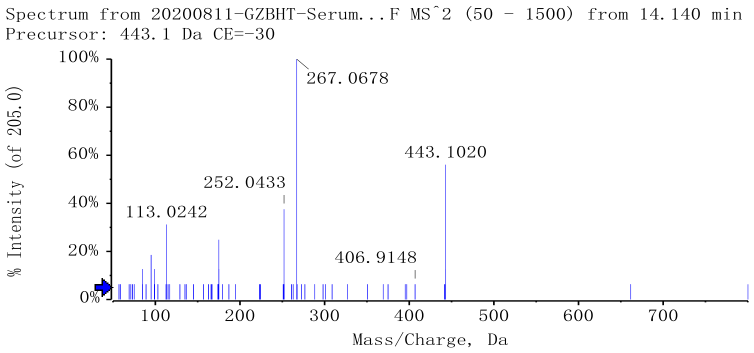

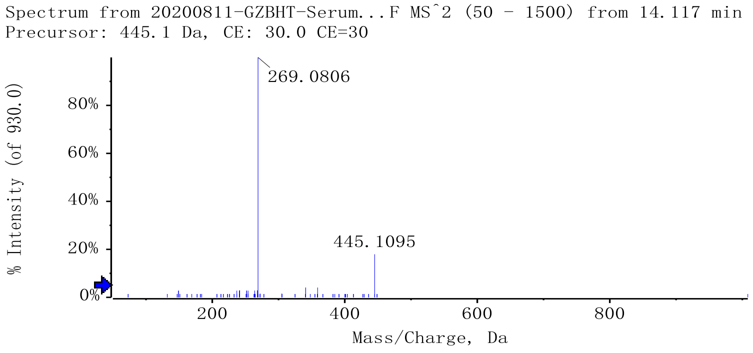

Figure S1. The chemical structure and corresponding MS/MS spectrum of absorbed components *in vivo.*

Chemical structure	Negative mode	Positive mode

P16-2 Sulfation of P16

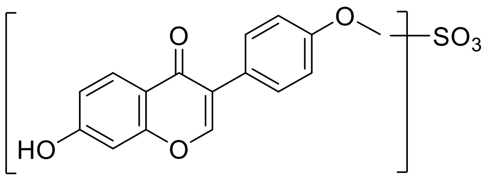

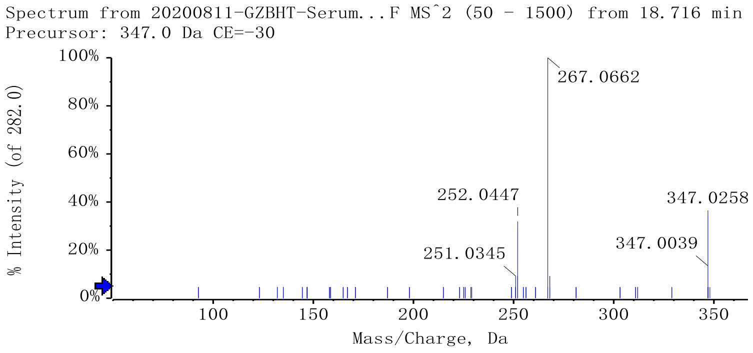

ND

P17 Glycyrrhizic acid

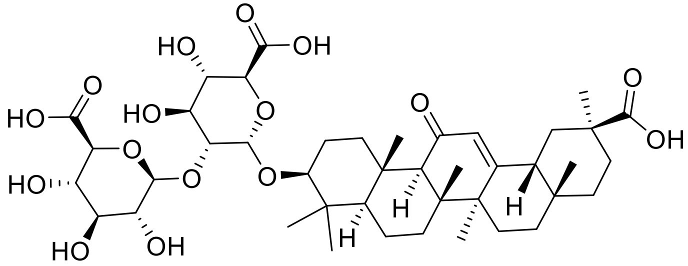

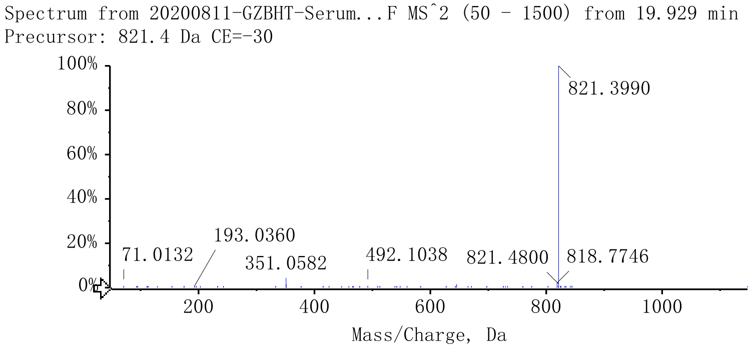

ND

P18 Timosaponin AIII

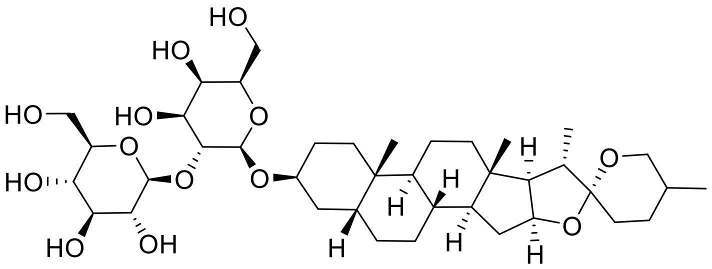

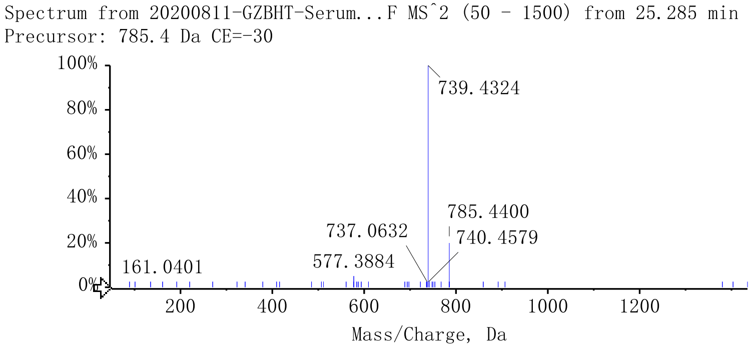

ND
