# Supplementary material for: Metabolic profiling and pharmacokinetic studies of Baihu-Guizhi decoction in rats by UFLC-Q-TOF–MS/MS and UHPLC-Q-TRAP-MS/MS
Source: Chin Med. 2022 Oct 4;17:117. doi: 10.1186/s13020-022-00665-w (PMC9531372; doi:10.1186/s13020-022-00665-w)
Supplement: Supplementary file 2 — Additional file 2: Figure S2. The chromatograms of mixed standards and serum sample after BHGZD gavage. [file 13020_2022_665_MOESM2_ESM.pdf]

Mixed standards

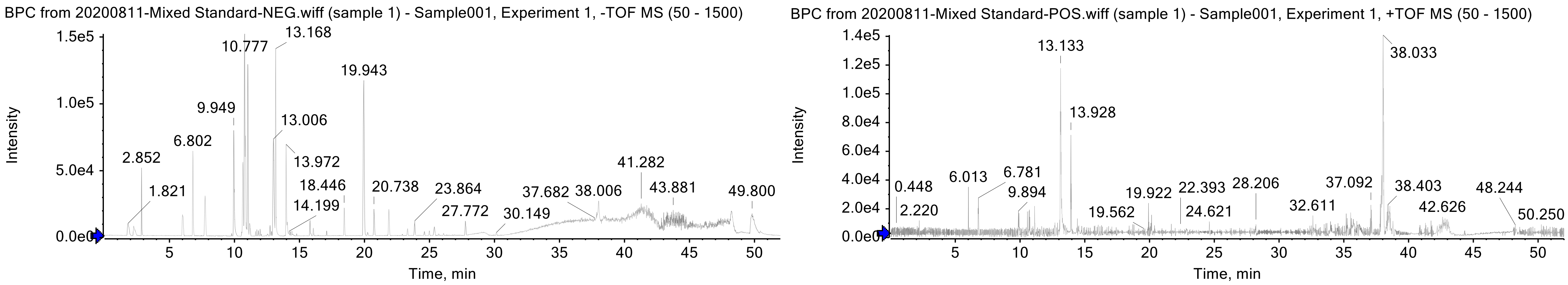

Serum sample after BHGZD gavage(2h)

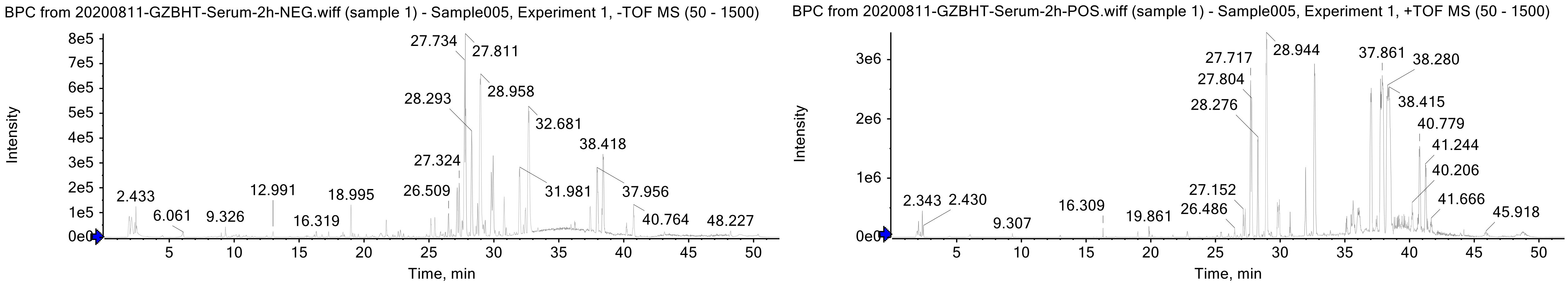

Figure S2. The chromatograms of mixed standards and serum sample after BHGZD gavage.
