# Supplementary material for: Metabolic profiling and pharmacokinetic studies of Baihu-Guizhi decoction in rats by UFLC-Q-TOF–MS/MS and UHPLC-Q-TRAP-MS/MS
Source: Chin Med. 2022 Oct 4;17:117. doi: 10.1186/s13020-022-00665-w (PMC9531372; doi:10.1186/s13020-022-00665-w)
Supplement: Supplementary file 3 — Additional file 3: Figure S3. The chromatogram of selectivity of the analytes in rat serum. [file 13020_2022_665_MOESM3_ESM.pdf]

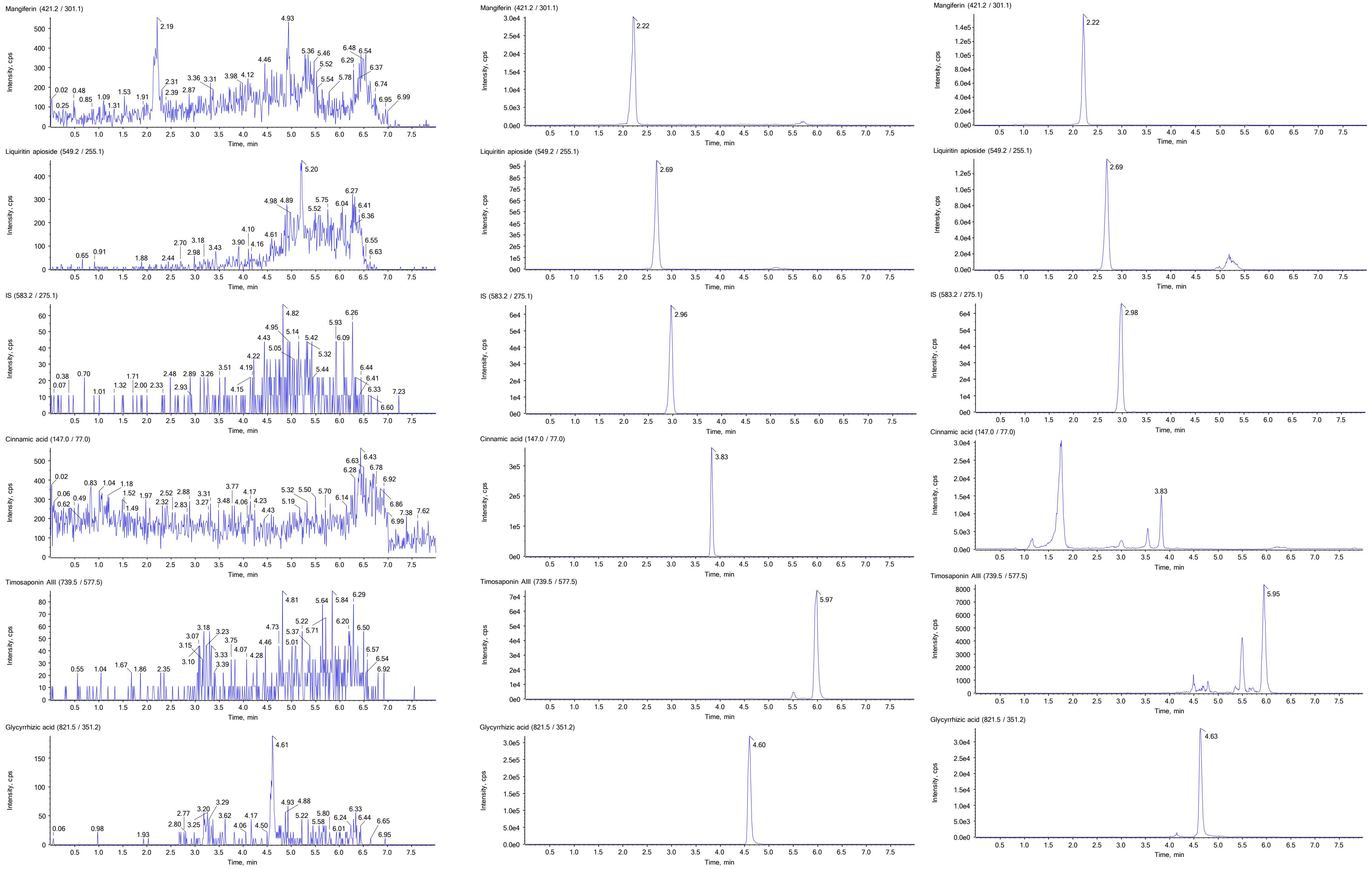

(a) **(b)** **(c)**

Figure S3. The chromatogram of selectivity of the analytes in rat serum: blank serum sample (a), blank serum sample spiked with standard compounds (b), and a serum sample obtained from a rat at 2h after BHGZD gavage (c).
