# Supplementary figures and images for: Metabolic profiling and pharmacokinetic studies of Baihu-Guizhi decoction in rats by UFLC-Q-TOF–MS/MS and UHPLC-Q-TRAP-MS/MS
Source: Chin Med. 2022 Oct 4;17:117. doi: 10.1186/s13020-022-00665-w (PMC9531372; doi:10.1186/s13020-022-00665-w)

Graphical abstract

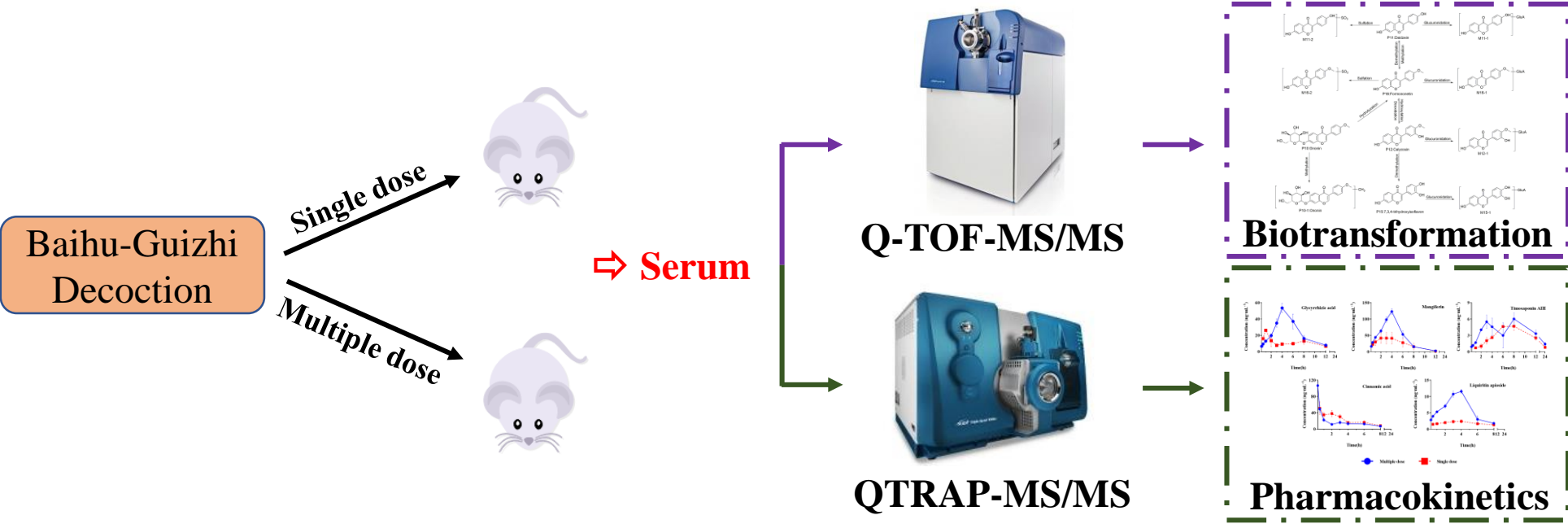

Supplement: Supplementary file 4 — Additional file 4: Figure S4. Graphical abstract. [file 13020_2022_665_MOESM4_ESM.pdf]
