# Supplementary material for: Metabolic profiling and pharmacokinetic studies of Baihu-Guizhi decoction in rats by UFLC-Q-TOF–MS/MS and UHPLC-Q-TRAP-MS/MS
Source: Chin Med. 2022 Oct 4;17:117. doi: 10.1186/s13020-022-00665-w (PMC9531372; doi:10.1186/s13020-022-00665-w)
Supplement: Supplementary file 5 — Additional file 5: Table S1. Identification of chemical profile of BHGZD in vitro by UFLC-Q-TOF-MS/MS. [file 13020_2022_665_MOESM5_ESM.pdf]

Table S1. Identification of chemical profile of BHGZD *in vitro* by UFLC-Q-TOF-MS/MS.

No.	Rt (mins)	Identification	Mass (Da)	Formula	Positive Ion (*m/z*)		Characteristic fragment ions in positive ion mode (*m/z*)	Negative Ion (*m/z*)		Characteristic fragment ions in negative ion mode (*m/z*)
					Mass [M+H]+	Error (ppm)		Mass [M-H]-	Error (ppm)	
1	1.35	Arginine [2]	174.1117	$C_6H_{14}N_4O_2$	175.1191	0.9	158.0908 [M+H-NH_3]+, 130.0974 [M+H-COOH]+, 116.0702 [M+H-CH_5N_3]+,70.0688 [M+H-HCOOH-CH_4N_3]+, 60.0603 [M+H-$C_5H_9NO_2$]+	—	—	
2	1.55	Proline [2]	115.0633	$C_5H_9NO_2$	116.0713	6.2	70.0688 [M+H-HCOOH]+,68.0534	—	—	
3	1.56	Pipecolic acid [2]	129.0790	$C_6H_{11}NO_2$	130.0867	3.4	84.0474 [M+H-HCOOH]+, 70.0691, 56.0551 [M+H-$C_3H_6O_2$]+	—	—	
4	2.01	Nicotinic acid [2]	123.0320	$C_6H_5NO_2$	124.0405	9.6	106.0337 [M+H-H_2O]+,78.0375 [M+H-HCOOH]+, 53.0451 [M+H-H_2O-C_3H_3N]+	—	—	
5	2.04	Citric acid[2]	192.0270	$C_6H_8O_7$	—	—		191.0212	7.6	129.0193 [M-H-OH-COOH]-, 111.0092 [M-H-OH-COOH-H_2O]-, 87.0104 [M-H-COOH-CH_2COOH]-, 85.0311, 67.0225, 57.0394 [C_3H_5O]-
6	2.16	Isoleucine [1,2]	131.0946	$C_6H_{13}NO_2$	132.1025	4.8	86.0999 [M+H-HCOOH]+,69.0738 [M+H-HCOOH-NH_3]+, 56.0548 [M+H-HCOOH-CH_4N]+	—	—	
7	3.14	Phenylalanine [2]	165.0790	$C_9H_{11}NO_2$	166.0866	1.8	120.0822 [M+H-HCOOH]+,103.0559 [M+H-HCOOH-NH_3]+, 77.0416 [M+H-HCOOH-C_2H_2-NH_3]+, 51.0281	—	—	
8	3.66	Pantothenic acid	219.1107	$C_9H_{17}NO_5$	220.1180	0.3	202.1045 [M+H-H_2O]+,174.1048 [M+H-HCOOH]+, 142.0860 [M+H-CH_3OH-HCOOH]+, 124.0765 [M+H-CH_3OH-HCOOH-H_2O]+, 98.0283 [M+H-CH_2COOH-CH_2OH-CH_3-OH]+, 90.0583 [M+H-$C_5H_{10}O_2$]+, 70.0314 [M+H-CH_2COOH-C_4H_9O-H_2O]+	218.1040	2.9	146.0784 [$C_6H_{12}NO_3$]-, 96.9602 [M-H-CH_2COOH-CH_2OH-CH_3-OH]-, 88.0405 [M-H-$C_5H_{10}O_2$]-, 71.0524 [M-H-$C_5H_{10}O_2$-OH]-
9	4.46	Protocatechuic acid [1,2]	154.0266	$C_7H_6O_4$	155.0343	2.7	137.0255 [M+H-H_2O]+, 109.0309 [M+H-HCOOH]+, 93.0372 [C_6H_5O]+, 65.0428 [C_5H_5]+	—	—	
10	5.05	Tryptophan [2]	204.0899	$C_{11}H_{12}N_2O_2$	205.0970	-0.7	188.0819 [M+H-NH_3]+, 146.0610 [$C_9H_{10}N_2$]+, 143.0736 [M+H-NH_2-HCOOH]+, 118.0663 [M+H-$C_2H_2NH_2COOH$]+, 91.0564 [M+H-C_4H_4-HCOOH-NH_2]+	203.0833	3.5	142.0646 [M-H-NH_2-COOH]-, 116.0491 [M-H-$C_2H_2NH_2COOH$]-, 74.0271 [$C_2H_4NO_2$]-
11	6.35	Benzaldehyde	106.0412	C_7H_6O	107.0501	8.7	91.0556, 77.0414 [M+H-HCHO]+, 65.0412 [C_5H_5]+, 51.0275	—	—	
12	6.71	p-Hydrobenzoic acid [1,2]	138.0317	$C_7H_6O_3$	139.0402	9.0	121.0308 [M+H-H_2O]+, 93.0368 [M+H-HCOOH]+, 77.0428 [M+H-OH-COOH]+, 65.0436 [M+H-OH-COOH-CH]+	137.0266	16.2	93.0358 [C_6H_5O]-, 65.0431 [M-H-COOH-C_2H_3]-
13	7.53	Neomangiferin [1,2]	584.1377	$C_{25}H_{28}O_{16}$	585.1449	-0.2	567.1313 [M+H-H_2O]+, 435.0911 [M+H-$C_5H_{10}O_5$]+, 387.0707 [M+H-H_2O-Glc]+, 369.0602 [M+H-3H_2O-Glc]+, 327.0497 [M+H-Glc-$C_5H_4O_2$]+, 303.0497 [M+H-Glc-$C_4H_8O_4$]+, 273.0497 [M+H-Glc-$C_5H_{10}O_5$]+	583.1304	-0.1	565.1227 [M-H-H_2O]-, 493.0999 [M-H-$C_3H_6O_3$]-, 463.0887 [M-H-$C_4H_8O_4$]-, 421.0798 [M-H-Glc]-, 403.0692 [M-H-Glc-H_2O]-, 331.0458 [M-H-Glc-$C_3H_6O_3$]-, 301.0353 [M-H-Glc-$C_4H_8O_4$]-, 259.0253 [M-2Glc]-
14	8.51	Caffeic acid [1,2]	180.0423	$C_9H_8O_4$	—	—		179.0367	9.5	135.0436 [$C_8H_7O_2$]-, 99.9995 [M-H-OH-C_2H_2COOH]-
15	9.64	p-Hydroxyphenyl-propionic acid [2]	166.0630	$C_9H_{10}O_3$	167.0706	1.9	121.0582 [M+H-HCOOH]+, 107.0454 [M+H-CH_3COOH]+, 84.9650 [M+H-C_6H_5O]+, 77.0411 [M+H-OH-C_2H_4COOH]+	—	—	
16	9.95	Mangiferin [1,2]	422.0849	$C_{19}H_{18}O_{11}$	423.0926	0.9	369.0615 [M+H-3H_2O]+, 339.0507 [M+H-3H_2O-CH_2O]+, 327.0506 [M+H-C_5H_2O]+, 315.0509 [M+H-$C_6H_4O_2$]+ 303.0497 [M+H-$C_4H_8O_4$]+, 273.0394 [M+H-$C_5H_{10}O_5$]+, 257.0450 [M+H-$C_5H_{10}O_6$]+	421.0780	0.8	331.0457 [M-H-$C_3H_6O_3$]-, 301.0351 [M-H-$C_4H_8O_4$]-, 271.0249 [M-H-$C_5H_{10}O_5$]-, 259.0253 [M-Glc]-, 243.0294 [M-H-Glc-OH]-
17	11.86	p-Coumaric acid [2]	164.0473	$C_9H_8O_3$	165.0550	2.4	147.0422 [M+H-H_2O]+, 119.0494 [M+H-HCOOH]+, 93.0337 [M+H-C_2H_3COOH]+, 91.0551 [M+H-HCOOH-CO]+, 65.0423 [C_5H_5]+	163.0422	13.2	119.0500 [C_8H_7O]-, 93.0360 [C_6H_5O]-, 65.0456 [C_5H_5]-
18	12.32	Isoschaftoside [2]	564.1479	$C_{26}H_{28}O_{14}$	565.1540	-2.1	511.1218 [M+H-3H_2O]+, 481.1125 [M+H-2H_2O-CHOH]+, 427.1018 [M+H-$C_4H_8O_4$-H20]+, 409.0911 [M+H-$C_4H_8O_4$-2H_2O]+ 391.0811 [M+H-$C_7H_{10}O_5$]+, 379.0814 [M+H-$C_4H_8O_4$-2H_2O-CHOH]+, 325.0704 [M+H-2$C_4H_8O_4$]+	563.1408	0.4	473.1101 [M-H-$C_3H_6O_3$]-, 443.1006 [M-H-$C_4H_8O_4$]-, 383.0773 [M-H-$C_4H_8O_4$-$C_2H_2O_2$]-, 353.0677 [M-H-$C_4H_8O_4$-$C_3H_6O_3$]-

Table S1. Identification of chemical profile of BHGZD *in vitro* by UFLC-Q-TOF-MS/MS.

No.	Rt (mins)	Identification	Mass (Da)	Formula	Positive Ion (*m/z*)		Characteristic fragment ions in positive ion mode (*m/z*)	Negative Ion (*m/z*)		Characteristic fragment ions in negative ion mode (*m/z*)
					Mass [M+H]+	Error (ppm)		Mass [M-H]-	Error (ppm)	
19	12.71	Orientin [2]	448.1006	$C_{21}H_{20}O_{11}$	449.1076	-0.4	431.0984 [M+H-H_2O]+, 413.0842 [M+H-2H_2O]+, 395.0774 [M+H-3H_2O]+, 329.0647 [M+H-$C_4H_8O_4$]+, 299.0575 [M+H-$C_5H_{10}O_5$]+	447.0937	0.9	357.0610 [M-H-$C_3H_6O_3$]-, 327.0531 [M-H-$C_4H_8O_4$]-,299.0534, 297.0412 [M-H-$C_5H_{10}O_5$]-, 284.0299 [M-H-Glu]-
20	13.15	Schaftoside [2]	564.1479	$C_{26}H_{28}O_{14}$	565.1546	-1.1	547.1380 [M+H-H_2O]+, 529.1333 [M+H-2H_2O]+, 511.1218 [M+H-3H_2O]+, 445.1068 [M+H-$C_4H_8O_4$]+, 397.0964 [M+H-$C_4H_8O_4$-H_2O-CHOH]+ 379.0814 [M+H-$C_4H_8O_4$-2H_2O-CHOH]+,	—	—	
21	13.62	Ferulic acid [1,2]	194.0579	$C_{10}H_{10}O_4$	—	—		193.0530	12.4	178.0240 [M-H-CH_3]-, 148.9005 [M-H-COOH]-, 133.0312 [M-H-COOH-CH_3]-
22	13.83	7-O-Methyl mangiferin [2]	436.1006	$C_{20}H_{20}O_{11}$	437.1075	-0.8	341.0661, 317.0646 [M+H-$C_4H_8O_4$]+, 287.0560 [M+H-$C_5H_{10}O_5$]+	435.0936	0.7	345.0624 [M-H-$C_3H_6O_3$]-, 330.0356 [M-H-$C_3H_6O_3$-CH_3]-, 315.0499 [M-H-$C_4H_8O_4$]-, 272.0306 [M-H-Glc]-
23	13.86	Liquiritigenin-7,4-diglucoside	580.1792	$C_{27}H_{32}O_{14}$	581.1865	0.2	419.1346 [M+H-Glc]+, 257.0821 [M+H-2Glc]+, 137.0246 [M+H-2Glc-C_8H_8O]+	579.1719	1.0	417.1198 [M-H-Glc]-, 255.0644 [M-H-2Glc]-, 135.0067 [M-H-2Glc-C_8H_8O]-
24	14.22	Vitexin [2]	432.1057	$C_{21}H_{20}O_{10}$	433.1133	0.9	415.1030 [M+H-H_2O]+, 397.0913 [M+H-2H_2O]+, 367.0820 [M+H-2H_2O-CHOH]+, 337.0719 [M+H-2H_2O-$C_2H_4O_2$]+, 313.0715 [M+H-$C_4H_8O_4$]+, 283.0611 [M+H-$C_5H_{10}O_5$]+	431.0988	0.9	341.0675 [M-H-$C_3H_6O_3$]-, 311.0571 [M-H-$C_4H_8O_4$]-, 283.0623
25	14.24	Licochalcone B	286.0841	$C_{16}H_{14}O_5$	—	—		285.0771	1.0	242.0889 [M-H-C_2H_3O]-, 109.0297, 93.0371 [C_6H_5O]-
26	14.28	Liquiritin [1,2]	418.1264	$C_{21}H_{22}O_9$	—	—		417.1195	1.1	255.0663 [M-H-Glc]-, 135.0091 [M-H-Glc-C_8H_8O]-, 119.0510 [M-H-Glc-C7H4O3]-, 91.0209 [M-H-Glc-C9H8O3]-
27	14.49	Liquiritin apioside [1,2]	550.1686	$C_{26}H_{30}O_{13}$	551.1754	-1.0	257.0820 [M+H-Api-Glc]+, 137.0240 [M+H-Api-Glc-C_8H_8O]+	549.1620	1.1	417.1209 [M-H-Api]-, 255.0659 [M-H-Api-Glc]-, 135.0092 [M-Api-Glc-C_8H_8O]-, 119.0510 [M-H-Api-Glc-$C_7H_4O_3$]-, 91.0209 [M-H-Api-Glc-$C_9H_8O_3$]-
28	14.78	Isovitexin [2]	432.1057	$C_{21}H_{20}O_{10}$	433.1134	1.1	397.0928 [M+H-2H_2O]+, 379.0822 [M+H-3H_2O]+, 367.0830 [M+H-2H_2O-CHOH]+, 337.0718 [M+H-2H_2O-$C_2H_4O_2$]+, 313.0717 [M+H-$C_4H_8O_4$]+, 283.0622 [M+H-$C_5H_{10}O_5$]+	431.0987	0.8	341.0676 [M-H-$C_3H_6O_3$]-, 311.0568 [M-H-$C_4H_8O_4$]-, 283.0612
29	16.32	Isoquercitrin	464.0955	$C_{21}H_{20}O_{12}$	465.1021	-1.4	447.0894 [M+H-H_2O]+, 369.0630 [M+H-$C_5H_5O_2$]+, 303.0513 [M+H-Glc]+, 273.0375 [M+H-Glc-CO]+	463.0886	0.8	331.0469 [M-H-$C_5H_{10}O_4$]-, 301.0351 [M-H-Glc]-, 259.0244 [M-H-Glc-C_2H_2O]-
30	16.84	Coumarin [2]	146.0368	$C_9H_6O_2$	147.0447	4.3	119.0466 [M+H-CO]+, 103.0556 [M+H-CO_2]+, 91.0564 [M+H-C_2O_2]+, 77.0416 [M+H-$C_3H_2O_2$]+, 65.0423 [M+H-$C_4H_2O_2$]+	—	—	
31	17.27	Hydroxygenkwanin [2]	300.0634	$C_{16}H_{12}O_6$	301.0709	0.9	283.0616 [M+H-H_2O]+, 167.0336 [M+H-$C_8H_8O_2$]+, 134.0376 [$C_8H_8O_2$]+	299.0571	3.3	239.0346 [M-H-OH-C_2H_3O]-, 199.0394
32	17.92	Genistin [2]	432.1057	$C_{21}H_{20}O_{10}$	433.1133	0.8	271.0605 [M+H-Glc]+, 153.0203 [M+H-Glc-C_8H_6O]+	431.0986	0.5	269.0411 [M-H-Glc]-, 151.0047 [M-Glc-C_8H_6O]-
33	18.94	Timosaponin N	936.4930	$C_{45}H_{76}O_{20}$	—	—		935.4863	0.6	917.4835 [M-OH]-, 755.4289 [M-H-OH-Glc]-
34	18.94	Timosaponin E	936.4930	$C_{45}H_{76}O_{20}$	—	—		935.4865	0.8	773.4419 [M-H-Glc]-, 611.3839 [M-H-Glc-Gal]-
35	19.46	Glycyroside	562.1686	$C_{27}H_{30}O_{13}$	563.1751	-1.4	269.0818 [M+H-Api-Glc]+	—	—	
36	19.85	Isoliquiritin apioside	550.1686	$C_{26}H_{30}O_{13}$	551.1759	-0.1	419.1346 [M+H-Api]+, 257.0815 [M+H-Api-Glc]+, 137.0245 [M+H-Api-Glc-C_8H_6O]+	549.1614	0.0	297.0764, 255.0662 [M-H-Api-Glc]-, 135.0245 [M-H-Api-Glc-C_8H_8O]-
37	19.95	Ononin [2]	430.1264	$C_{22}H_{22}O_9$	431.1335	-0.4	269.0812 [M+H-Glc]+, 254.0580 [M+H-Glc-CH_3]+	—	—	
38	20.37	Isoliquiritin [2]	418.1264	$C_{21}H_{22}O_9$	419.1337	0.9	257.0817 [M+H-Glc]+, 147.0446 [M+H-Glc-$C_6H_5O_2$]+, 137.0242 [M+H-Glu-C_8H_8O]+, 119.0495 [M+H-Glu-$C_7H_6O_3$]+	417.1191	0.3	255.0662 [M-H-Glc]-, 148.0163, 135.0085 [M-H-Glc-C_8H_8O]+, 119.0503 [M-H-$C_7H_4O_3$]-
39	20.86	Timosaponin E1	936.4930	$C_{45}H_{76}O_{20}$	—	—		935.4859	0.1	773.4401 [M-H-Glc]-, 611.3850 [M-H-Glc-Gal]-
40	20.88	Officinalisnin I	920.4981	$C_{45}H_{76}O_{19}$	921.4954	-10.8	759.4396 [M+H-Glc]+, 597.3895 [M+H-Glc-Gal]+, 433.3319 [M+H-Glc-Gal-Glc]+, 415.3207 [M+H-Glc-Gal-Glc-H_2O]+, 271.2051	—	—	
41	20.96	Neoisoliquiritin [1,2]	418.1264	$C_{21}H_{22}O_9$	419.1337	0.2	257.0824 [M+H-Glc]+, 137.0243 [$C_7H_5O_3$]+, 119.0507 [M+H-Glc-$C_7H_5O_3$]+	417.1191	0.2	255.0663 [M-H-Glc]-, 148.0614 [M-H-Glc-C_7H_6O]-, 135.0087 [M-H-Glc-C_8H_8O]-, 119.0510 [M-H-$C_7H_4O_3$]-, 91.0207 [M-H-Glc-$C_9H_7O_3$]-

Table S1. Identification of chemical profile of BHGZD *in vitro* by UFLC-Q-TOF-MS/MS.

No.	Rt (mins)	Identification	Mass (Da)	Formula	Positive Ion (*m/z*) Mass [M+H]+	Positive Ion (*m/z*) Error (ppm)	Characteristic fragment ions in positive ion mode (*m/z*)	Negative Ion (*m/z*) Mass [M-H]-	Negative Ion (*m/z*) Error (ppm)	Characteristic fragment ions in negative ion mode (*m/z*)
42	21.39	Liquiritigenin [1,2]	256.0736	$C_{15}H_{12}O_4$	—	—		255.0663	0.6	135.0084 [M-H-C_8H_8O]-, 119.0509 [M-H-$C_7H_4O_3$]-, 91.0196 [M-H-$C_9H_8O_3$]-
43	21.96	Cinnamic acid [1,2]	148.0524	$C_9H_8O_2$	149.0601	2.4	131.0498 [M+H-H_2O]+, 103.0552 [M+H-HCOOH]+, 77.0417 [C_6H_5]+, 51.0289 [M+H-C_4H_4-HCOOH]+	147.0470	12.8	103.0539 [M-COOH]-, 77.0383 [C_6H_5]-, 61.9927
44	22.89	Timosaponin B II [1,2]	920.4981	$C_{45}H_{76}O_{19}$	—	—		919.4932	2.6	757.4462 [M-H-Glc]-, 595.3910 [M-H-Glc-Gal]-
45	22.93	Xilingsaponin B	902.4875	$C_{45}H_{74}O_{18}$	903.4939	-1.0	741.4363 [M+H-Glc]+, 579.3856 [M+H-2Glc]+, 417.3345 [M+H-2Glc-Gal]+, 399.3247 [M+H-2Glc-Gal-H_2O]+, 273.2204 [M+H-2Glc-Gal-H_2O-$C_8H_{14}O$]+, 255.2099 [M+H-2Glc-Gal-H_2O-$C_8H_{15}O_2$]+, 163.0804 [M+H-2Glc-Gal-H_2O-$C_{14}H_{21}O_2$]+, 145.0500	—	—	
46	24.98	Timosaponin D	918.4824	$C_{45}H_{74}O_{19}$	919.4888	-1.0	757.4351 [M+H-Glc]+, 595.3837 [M+H-Glc-Gal]+, 433.3320 [M+H-Glc-Gal-Glc]+, 289.2172 [M+H-Glc-Gal- Glc-$C_8H_{14}O$-H_2O]+, 271.2060 [M+H-Glc-Gal- Glc-$C_8H_{14}O$-2H_2O]+, 163.0605 [M+H-Glc-Gal- Glc-$C_{16}H_{28}O_2$-H_2O]+	917.4759	0.8	755.4307 [M-H-Glc]-
47	26.54	Genkwanin [2]	284.0685	$C_{16}H_{12}O_5$	—	—		283.0628	5.6	268.0386 [M-H-CH_3]-, 224.0485 [M-H-OCH_3-CO]-, 211.0385, 135.0083 [M-H-$C_9H_8O_2$]-, 91.0188 [M-H-$C_{10}H_8O_4$]-
48	27.10	Glypallichalcone	284.1049	$C_{17}H_{16}O_4$	—	—		283.0986	3.7	268.0382 [M-H-CH_3]-, 221.0966 [M-H-OCH_3-OCH_3]-, 133.0656 [M-H-$C_9H_{10}O_2$]-
49	28.96	Pseudoproto-timosaponin AIII [1,2]	902.4875	$C_{45}H_{74}O_{18}$	903.4937	-1.2	741.4362 [M+H-Glc]+, 579.3856 [M+H-Glc-Gal]+, 417.3344 [M+H-Glc-Gal-Glc]+, 399.3249 [M+H-Glc-Gal-Glc-H_2O]+, 273.2211 [M+H-Glc-Gal-Glc-H_2O-$C_8H_{14}O$]+, 163.0804 [M+H-Glc-Gal-Glc-H_2O-$C_{14}H_{21}O_2$]+,	901.4811	1.0	739.4352 [M-H-Glc]-
50	30.27	Isoliquiritigenin [1,2]	256.0736	$C_{15}H_{12}O_4$	—	—		255.0663	3.0	135.0079 [M-H-C_8H_8O]-, 119.0494 [M-H-$C_7H_4O_3$]-, 91.0240 [M-H-$C_9H_8O_3$]-
51	30.38	Formononetin [2]	268.0736	$C_{16}H_{12}O_4$	269.0810	0.7	253.0512 [M-CH_3]+, 237.0542 [M-OCH_3]+, 226.0628 [M-C_2H_2O]+, 213.0912 [M+H-C_3H_4O]+, 197.0612, 181.0646, 169.0640 [M+H-CHO-2CO-CH_3]+, 118.0435 [M+H-$C_7H_4O_3$-CH_3]+	267.0677	5.3	252.0424 [M-H-CH_3]-, 251.0336 [M-H-O]-, 223.0402 [M-H-O-CO]-, 195.0451 [M-H-O-2CO]-, 167.0493 [M-H-CHO-2CO-CH_3]-, 132.0218 [M-H-$C_7H_3O_3$]-, 91.0188 [M-H-$C_9H_5O_3$-CH_3]-
52	32.03	Glabrolide [2]	468.3240	$C_{30}H_{44}O_4$	469.3310	-0.4	451.3201 [M+H-H_2O]+, 439.3194 [M+H-CHOH]+, 217.1597, 189.1633	—	—	
53	33.20	Anemarrhenasaponin III	756.4296	$C_{39}H_{64}O_{14}$	—	—		755.4226	0.4	593.3697 [M-H-Glc]-, 161.0428 [$C_6H_9O_5$]-
54	33.33	Anemarrhenasaponin I [1,2]	758.4453	$C_{39}H_{66}O_{14}$	—	—		757.4374	-0.8	595.3901 [M-H-Glc]-, 161.0428 [$C_6H_9O_5$]-
55	33.35	Smilageninoside	740.4347	$C_{39}H_{64}O_{13}$	741.4407	-1.7	417.3358 [M+H-Glc-Man]+, 399.3256 [M+H-Glc-Man-H_2O]+, 381.3148 [M+H-Glc-Man-2H_2O]+, 271.2057 [M+H-Glc-Man-$C_8H_{18}O_2$]+, 253.1951 [M+H-Glc-Man-$C_8H_{18}O_2$-H_2O]+, 145.0507 [[M+H-Glc-Man-$C_{16}H_{28}O_2$-H_2O]+	—	—	
56	33.63	Glycycoumarin	368.1260	$C_{21}H_{20}O_6$	369.1333	0.0	313.0706 [M+H-C_4H_8]+, 285.0760 [M+H-C_5H_9-CH_3]+, 271.0594 [M+H-C_6H_8-H_2O]+, 243.0654 [M+H-$C_6H_4O_2$-H_2O]+, 211.0381	367.1194	1.7	335.0916, 309.0390, 298.0482 [M-H-C_5H_9]-, 283.0228 [M-H-C_5H_9-CH_3]-, 255.0294 [M-H-$C_7H_{12}O$]-
57	34.26	Glycyrrhizic acid [1,2]	822.4038	$C_{42}H_{62}O_{16}$	823.4085	-3.1	647.3766 [M+H-GlcA]+, 471.3766 [M+H-2GlcA]+, 453.3361 [M+H-2GlcA-H_2O]+	821.3981	2.0	351.0576 [M-H-$C_{30}H_{46}O_4$]-, 193.0353 [$C_5H_8O_5$COOH]-, 175.0251 [$C_5H_6O_4$COOH]-, 113.0253 [M-H-2GlcA-$C_{24}H_{35}O_2$]-
58	34.28	Glycyrrhetic acid-3-O-β-D-glucuronide	646.3717	$C_{36}H_{54}O_{10}$	647.3778	-1.8	471.3439 [M+H-C_5H_7COOH]+, 453.3363 [M+H-$C_6H_8O_7$]+, 435.3250 [M+H-$C_6H_8O_7$-H_2O]+, 407.3289 [M+H-$C_6H_8O_7$-HCOOH]+	—	—	
59	35.15	Timosaponin G	756.4296	$C_{39}H_{64}O_{14}$	—	—		755.4231	1.0	593.3715 [M-H-Glc]-, 161.0455 [$C_6H_9O_5$]-
60	35.23	Yamogenin	414.3134	$C_{27}H_{42}O_3$	415.3203	-0.8	397.3093 [M+H-H_2O]+, 283.2418, 271.2072 [M+H-$C_8H_{14}O_2$]+, 253.1952 [M+H-$C_{11}H_{14}O$]+, 159.1166, 143.0850 [M+H-$C_{18}H_{24}O_2$]+, 69.0730 [C_5H_9]+	—	—	
61	35.23	Neogitogenin	432.3240	$C_{27}H_{44}O_4$	433.3313	0.2	397.3099 [M+H-H_2O]+, 283.2418, 271.2073 [M+H-$C_8H_{14}O_2$]+, 253.1958 [M+H-$C_{11}H_{14}O$]+, 159.1177	—	—	

Table S1. Identification of chemical profile of BHGZD *in vitro* by UFLC-Q-TOF-MS/MS.

No.	Rt (mins)	Identification	Mass (Da)	Formula	Positive Ion (*m/z*)		Characteristic fragment ions in positive ion mode (*m/z*)	Negative Ion (*m/z*)		Characteristic fragment ions in negative ion mode (*m/z*)
					Mass [M+H]+	Error (ppm)		Mass [M-H]-	Error (ppm)	
62	36.88	cis-Hinokiresinol	252.1150	$C_{17}H_{16}O_2$	—	—		251.1086	3.3	235.0755 [M-H-OH]-, 157.0648 [M-H-C_6H_5O]-, 117.0335 [M-H-$C_9H_{10}O$]-, 93.0353 [M-H-$C_{11}H_{10}O$]-
63	36.89	Biochanin A	284.0685	$C_{16}H_{12}O_5$	—	—		283.0628	5.6	268.0368 [M-H-CH_3]-, 211.0388 [M-H-COH-OCH_3]-, 239.0294 [M-H-C_2H_3O]-
64	37.17	Licoisoflavone A	354.1103	$C_{20}H_{18}O_6$	355.1179	0.7	337.1033 [M+H-H_2O]+, 201.0922 [$C_{13}H_{13}O_2$]+, 189.0924, 179.0339 [M+H-$C_{11}H_{12}O_2$]+, 151.0393, 127.0409 [$C_6H_7O_3$]+, 123.0451 [M+H-$C_9H_5O_4$-C_4H_7]+	353.1035	1.2	227.0706 [$C_{14}H_{14}O_3$]-, 150.0316 [M-H-$C_{10}H_4O_4$-CH_3]-, 125.0238 [$C_6H_5O_3$]-
65	37.33	Semilicoisoflavone B	352.0947	$C_{20}H_{16}O_6$	353.1018	-0.3	311.0548 [M+H-C_3H_6]+, 153.0193 [M+H-$C_{13}H_{12}O_2$]+	351.0875	0.2	283.0980 [M-H-C_5H_8]-, 265.0891 [$C_{15}H_5O_5$]-, 241.0884, 199.0768 [M-H-$C_7H_4O_4$]-
66	37.63	Timosaponin A II	756.4296	$C_{39}H_{64}O_{14}$	757.4363	-0.7	595.3844 [M+H-Glc]+, 433.3333 [M+H-Glc-Gal]+, 289.2172 [M+H-Glc-Gal-$C_9H_{13}O_2$]+, 163.0616 [$C_6H_{11}O_5$]+	755.4221	-0.3	593.3731 [M-H-Glc]-, 161.0443 [$C_6H_9O_5$]-
67	37.67	Diosgenin [2]	414.3134	$C_{27}H_{42}O_3$	415.3206	-0.1	397.3090 [M+H-H_2O]+, 301.2545 [M+H-$C_6H_{10}O_2$]+, 271.2053 [M+H-$C_8H_{16}O_2$]+, 253.1958 [M+H-$C_{11}H_{14}O$]+, 69.0738 [C_5H_9]+	—	—	
68	37.67	Markogenin	432.3240	$C_{27}H_{44}O_4$	433.3309	-0.8	289.2177 [M+H-$C_8H_{16}O_2$]+, 271.2053, 253.1964 [M+H-$C_{11}H_{14}O$]+, 161.1332, 145.1023 [$C_8H_{17}O_2$]+, 69.0738 [C_5H_9]+	—	—	
69	38.11	Licoflavonol	354.1103	$C_{20}H_{18}O_6$	355.1174	-0.5	299.0546 [M+H-C_4H_8]+, 165.0209	353.1035	1.2	297.0391 [M-H-C_4H_8]-, 285.1113 [M-C_5H_9]-, 151.0768 [M-H-$C_{11}H_6O_4$]-, 133.0286 [M-H-$C_{12}H_{12}O_4$]-
70	38.27	Glycyrrhisoflavanone	368.1260	$C_{21}H_{20}O_6$	369.1336	0.9	313.0723 [M+H-C_3H_4O]+, 285.0766 [M+H-C_5H_8O]+ 270.0525 [M+H-C_5H_8O-CH_3]+, 257.0806, 243.0659, 123.0442 [M+H-$C_{14}H_{14}O_4$]+	367.1195	2.2	309.0406 [M-H-C_3H_6O]-, 297.0406 [M-H-C_5H_{10}]-,284.0321, 201.0184 [M-H-$C_8H_6O_4$]-
71	38.42	Uralenin	356.1260	$C_{20}H_{20}O_6$	357.1334	0.3	301.0709 [M+H-$C_2H_2O_2$]+, 283.0596, 179.0378 [M+H-$C_{11}H_{14}O_2$]+, 175.0387, 147.0451, 153.0527 [M+H-$C_{13}H_{16}O_2$]+,	355.1185	-0.5	229.0856 [M-H-C_3H_6-$C_4H_4O_2$]-, 174.0322, 125.0238 [$C_6H_5O_3$]-
72	38.71	Licoisoflavone B	352.0947	$C_{20}H_{16}O_6$	353.1023	1.0	335.0907 [M+H-H_2O]+, 311.0551 [M+H-C_3H_6]+, 307.0980, 227.0706 [M+H-$C_6H_4O_2$-H_2O]+, 153.0188 [M+H-$C_{13}H_{12}O_2$]+	351.0881	2.0	336.0603 [M-H-CH_3]-, 333.0780 [M-H-H_2O]-, 321.0394 [M-H-C_2H_6]-
73	38.84	Glyasperin C	356.1624	$C_{21}H_{24}O_5$	357.1699	0.8	301.1079 [M+H-C_4H_8]+, 221.1173 [M+H-$C_8H_8O_2$]+, 179.0710 [M+H-$C_6H_5O_2$-C_5H_9]+, 165.0559, 153.0549 [M+H-$C_{13}H_{16}O_2$]+, 137.0602 [M+H-$C_{13}H_{16}O_3$]+, 123.0451 [M+H-$C_{14}H_{18}O_3$]+	355.1561	2.7	323.1271 [M-H-CH_3-OH]-, 233.1179 [M-H-$C_7H_6O_2$]-, 207.1014 [M-H-$C_9H_8O_2$]-, 147.0442, 135.0454 [M-H-$C_{13}H_{16}O_3$]-, 109.0307 [M-H-$C_{15}H_{18}O_3$]-
74	39.29	Glyasperin F	354.1103	$C_{20}H_{18}O_6$	—	—		353.1040	2.8	338.1161 [M-H-CH_3]-, 298.0449, 218.0932 [M-H-$C_7H_3O_3$]-, 151.0735 [M-H-$C_{13}H_{14}O_2$]-, 133.0636
75	39.65	Gancaonin Z	340.1311	$C_{21}H_{24}O_4$	—	—		339.1246	2.5	219.0653 [M-H-$C_7H_4O_2$]-, 183.0096, 133.0640 [C_9H_9O]-, 119.5000 [M-H-$C_9H_{10}O_2$-C_5H_{10}]-
76	39.81	Phaseollinisoflavan	324.1362	$C_{20}H_{20}O_4$	325.1436	0.6	269.0754, 189.0902 [M+H-$C_8H_8O_2$]+, 171.0814 [M+H-$C_8H_8O_2$-H_2O]+, 123.0445 [M+H-$C_{13}H_{14}O_2$]+	—	—	
77	39.93	Inflacoumarin A	322.1205	$C_{20}H_{18}O_4$	—	—		321.1145	3.9	306.0861 [M-H-CH_3]-, 175.0796 [M-H-$C_9H_6O_2$]-, 145.0270 [M-H-$C_{11}H_{12}O_2$]-, 107.0512 [M-H-$C_{11}H_{12}O_3$]-
78	40.01	Gancaonin C	354.1103	$C_{20}H_{18}O_6$	355.1174	-0.5	299.0554 [M+H-C_3H_4O]+, 287.0560 [M+H-C_4H_4O]+, 243.0695 [M+H-C_6H_6O-H_2O]+, 69.0737 [C_5H_9]+	353.1030	-0.2	297.0392 [M-H-C_3H_4O]-, 269.0469 [M-H-C_5H_8O]-
79	40.76	Glycyrrhisoflavone	354.1103	$C_{20}H_{18}O_6$	355.1179	0.9	337.1070 [M+H-H_2O]+, 201.0919, 189.0914 [M+H-$C_{10}H_{14}O_2$]+, 179.0344 [M+H-$C_{11}H_{14}O_2$]+, 153.0539 [M+H-$C_{13}H_{14}O_2$]+, 151.0393 [M+H-$C_{11}H_{18}O_4$]+, 123.0441 [M+H-$C_9H_5O_4$-C_4H_7]+	353.1042	3.2	228.0772 [M-H-$C_6H_5O_3$]-, 125.0248 [M-H-$C_{14}H_{12}O_3$]-, 57.0397 [M-H-$C_{18}H_{16}O_4$]-
80	40.96	Licoflavone A	322.1205	$C_{20}H_{18}O_4$	—	—		321.1141	2.6	306.0888 [M-H-CH_3]-, 261.0918, 248.0794 [M-H-C_4H_8-OH]-, 213.0794 [M-H-CH_3-C_6H_5O]-
81	41.10	Timosaponin A IV	740.4347	$C_{39}H_{64}O_{13}$	741.4407	-1.8	579.3901 [M+H-Glc]+, 417.3374 [M+H-Glc-Man]+, 399.3266 [M+H-2Glc-Man-OH]+, 271.2072, 253.1961 [M+H-$C_{30}H_{48}O_5$]+, 145.0503 [$C_6H_9O_4$]+	739.4277	0.4	577.3766 [M-H-Glu]-, 161.0452 [$C_6H_9O_5$]-

Table S1. Identification of chemical profile of BHGZD *in vitro* by UFLC-Q-TOF-MS/MS.

No.	Rt (mins)	Identification	Mass (Da)	Formula	Positive Ion (*m/z*)		Characteristic fragment ions in positive ion mode (*m/z*)	Negative Ion (*m/z*)		Characteristic fragment ions in negative ion mode (*m/z*)
					Mass [M+H]+	Error (ppm)		Mass [M-H]-	Error (ppm)	
83	41.26	Licoricone	382.1416	$C_{22}H_{22}O_6$	383.1493	0.9	327.0866 [M+H-C_4H_7]+, 299.0908 [M+H-C_5H_9-CH_3]+, 191.0727 [M+H-$C_9H_5O_3$-OCH_3]+, 178.0643	381.1347	0.9	351.0884 [M-OCH_3]-, 323.0568
84	41.42	Isolicoflavonol	354.1103	$C_{20}H_{18}O_6$	355.1178	0.5	299.0562 [M+H-C_4H_8]+, 243.0662, 217.0495 [M+H-$C_9H_{14}O$]+, 153.0194 [M+H-$C_{13}H_{14}O_2$]+, 147.0430 [M+H-$C_9H_5O_5$-CH_3]+, 123.0450 [M+H-$C_{14}H_{16}O_3$]+	353.1040	2.6	284.0326 [M-H-C_5H_9]-, 243.1010 [M-H-$C_7H_{10}O$]-, 216.0422, 201.0910 [M-H-$C_7H_4O_4$]-
85	42.91	Timosaponin A III [1,2]	740.4347	$C_{39}H_{64}O_{13}$	741.4403	-2.3	579.3868 [M+H-Glc]+, 417.3369 [M+H-Glc-Gal]+, 399.3255 [M+H-Glc-Gal-H_20]+, 273.2220, 255.2111 [$C_9H_{19}O_8$]+, 163.0607 [$C_6H_{11}O_5$]+, 145.0502 [$C_6H_9O_4$]+	739.4275	0.1	277.3790 [M-H-Glc]-, 161.0454 [$C_6H_9O_5$]-
86	42.92	Timosaponin A I	578.3819	$C_{33}H_{54}O_8$	579.3886	-0.9	417.3375 [M+H-Glc]+, 273.2224 [M+H-$C_{20}H_{34}O_2$]+, 255.2119 [$C_9H_{19}O_8$]+	—	—	
87	42.93	Sarsasapogenin [2]	416.3291	$C_{27}H_{44}O_3$	417.3362	-0.3	273.2225 [M+H-$C_8H_{16}O_2$]+, 255.2121 [M+H-$C_8H_{16}O_2$-H_2O]+, 173.1335, 147.1181, 145.1025 [$C_8H_{17}O_5$]+, 69.0740 [C_5H_9]+	—	—	
88	42.94	Licoflavone C	338.1154	$C_{20}H_{18}O_5$	—	—		337.1094	3.7	293.0432 [M-H-C_2H_4O]-, 281.0472 [M-H-C_4H_8]-
89	43.12	Glabrocoumarin	336.0998	$C_{20}H_{16}O_5$	337.1069	-0.4	295.0618 [M+H-C_3H_6]+, 283.0625, 201.0548 [M+H-$C_7H_4O_3$]+, 137.0232 [M+H-$C_{13}H_{12}O_2$]+	335.0926	0.4	320.0704 [M-H-CH_3]-, 291.1007 [M-H-CO_2]-, 135.0094 [M-H-$C_{13}H_{12}O_2$]-
90	43.67	Glabridin [2]	324.1363	$C_{20}H_{20}O_4$	325.1437	0.8	189.0909 [M+H-$C_8H_8O_2$]+, 149.0596, 147.0800, 123.0453 [M+H-$C_{13}H_{14}O_2$]+	323.1301	3.6	201.0895 [M-H-$C_7H_6O_2$]-, 187.0754 [M-H-$C_8H_8O_2$]-, 135.0440 [M-H-$C_{12}H_{12}O_2$]-, 121.0289 [M-H-$C_{13}H_{14}O_2$]-
91	44.42	Glepidotin B	340.1311	$C_{20}H_{20}O_5$	—	—		339.1249	3.3	219.0651 [M-H-$C_8H_8O_2$]-, 119.0504 [M-H-$C_{12}H_{12}O_4$]-
92	44.76	Gancaonin A	352.1311	$C_{21}H_{20}O_5$	353.1386	0.6	189.0901 [M+H-C_4H_8-C_7H_8O]+, 177.0585 [M+H-$C_{11}H_{12}O_2$]+, 171.0816, 153.0556 [M+H-$C_{12}H_8O_3$]+, 147.0803 [M+H-$C_{12}H_{14}O_3$]+	—	—	
93	44.88	Glepidotin A	338.1154	$C_{20}H_{18}O_5$	—	—		337.1100	5.6	
94	46.12	Glycyrin	382.1416	$C_{22}H_{22}O_6$	383.1489	0.0	299.0912 [M+H-C_5H_9-CH_3]+, 284.0677 [M+H-C_5H_9-CH_3-CH_3]+	—	—	
95	47.55	Glabrone [2]	336.0998	$C_{20}H_{16}O_5$	337.1073	0.8	295.0616 [M+H-C_3H_6]+, 153.0185 [M+H-$C_{11}H_{14}O_3$]+	335.0934	2.7	319.0615 [M-H-OH]-, 305.0464 [M-H-C_2H_6]-
96	47.72	Glyasperin B	370.1416	$C_{21}H_{22}O_6$	371.1490	0.2	315.0870 [M+H-C_4H_7]+, 297.0753 [M+H-C_4H_7-OH]+, 193.0475 [M+H-C_5H_9-$C_6H_5O_2$]+, 123.0444 [$C_7H_6O_2$]+	369.1349	1.4	207.1000 [$C_{12}H_{15}O_3$]-, 192.0773, 161.0234 [M-H-C_5H_9-OCH_3-$C_6H_5O_2$]-, 149.0220 [M-H-$C_{13}H_{16}O_3$]-, 137.0223 [$C_8H_9O_2$]-, 124.0156
97	48.26	Gancaonin B	368.1260	$C_{21}H_{20}O_6$	369.1332	-0.2	313.0701 [M+H-C_4H_7]+, 295.0582 [M+H-C_4H_7-OH]+, 179.0338 [M+H-C_5H_9-$C_7H_7O_2$]+, 149.0239 [M+H-$C_{12}H_{12}O_4$]+	—	—	
98	48.38	Licoagroisoflavone	336.0998	$C_{20}H_{16}O_5$	337.1074	1.1	321.0742 [M+H-CH_3]+, 319.0999 [M+H-OH]+	—	—	
99	48.53	Glyasperin D	370.1780	$C_{22}H_{26}O_5$	371.1856	0.8	315.1242 [M+H-C_4H_7]+, 303.1232 [M+H-C_4H_7-CH_3]+, 235.1339 [M+H-$C_8H_8O_2$]+, 193.0857 [M+H-C_4H_7-CH_3-$C_6H_5O_2$]+, 181.0859, 167.0713 [M+H-$C_{13}H_{16}O_2$]+, 123.0453 [M+H-$C_{15}H_{20}O_3$]+, 69.0737 [C_5H_9]+	369.1719	3.1	217.0826 [M-H-$C_8H_8O_3$]-, 147.0443, 135.0434 [M-H-$C_{14}H_{18}O_3$]-
100	49.00	Anemarrhenasaponin Ia	772.4609	$C_{46}H_{68}O_{14}$	—	—		771.4445	-11.8	725.4410 [M-H-CH_2OH-CH_3]-, 563.3923 [M-H-Glc-OCH_3]-
101	52.74	Glyurallin A	352.1311	$C_{21}H_{20}O_5$	353.1383	0.0	297.0762 [M+H-C_4H_7]+, 267.0630 [M+H-C_4H_7-OCH_3]+	—	—	
102	52.79	β-Glycyrrhetnic acid [2]	470.3396	$C_{30}H_{46}O_4$	471.3468	-0.2	425.3403 [M+H-COOH]+, 407.3302 [M+H-COOH-OH]+, 317.2114 [M+H-$C_{10}H_{18}O$]+, 235.1708 [M+H-$C_{15}H_{24}O_2$]+, 189.1649 [M+H-$C_{15}H_{24}O_2$-COOH]+	469.3327	0.8	425.3410 [M-H-COOH]-, 355.2645 [M-H-$C_6H_{10}O_2$]-
103	69.00	Stigmasterol	412.3705	$C_{29}H_{48}O$	413.3810	7.8	367.3759 [M+H-OH-C_2H_5]+, 350.3483, 301.1405 [M+H-C_8H_{16}]+, 69.0743 [C_5H_9]+	—	—	
