# Supplementary material for: Metabolic profiling and pharmacokinetic studies of Baihu-Guizhi decoction in rats by UFLC-Q-TOF–MS/MS and UHPLC-Q-TRAP-MS/MS
Source: Chin Med. 2022 Oct 4;17:117. doi: 10.1186/s13020-022-00665-w (PMC9531372; doi:10.1186/s13020-022-00665-w)
Supplement: Supplementary file 6 — Additional file 6: Table S2. Quantification analysis of calcium in Gypsum Fibrosum and full prescription BHGZD by ICP-AES. [file 13020_2022_665_MOESM6_ESM.pdf]

Table S2 Quantification analysis of calcium in Gypsum Fibrosum and full prescription BHGZD by ICP-AES.

Sample	Concentration of calcium (n=2, Mean ± SD, μg/mL)	Normalized Concentration (μg/mL)	Final Concentration of calcium[a] (μg/mL)
Blank	0.716 ± 0.04	-	-
Gypsum Fibrosum	6.255 ± 0.15	5.539	553.9
BHGZD	8.980 ± 0.41	8.264	826.4

a. Sample preparation: Gypsum Fibrosum solution or full prescription BHGZD diluted 100x before ICP-AES detection.
